# NAKED ENDOSPERM1, NAKED ENDOSPERM2, and OPAQUE2 interact to regulate gene networks in maize endosperm development

**DOI:** 10.1093/plcell/koad247

**Published:** 2023-10-05

**Authors:** Hao Wu, Mary Galli, Carla J Spears, Junpeng Zhan, Peng Liu, Ramin Yadegari, Joanne M Dannenhoffer, Andrea Gallavotti, Philip W Becraft

**Affiliations:** Genetics, Development and Cell Biology Department, Iowa State University, Ames, IA 50011, USA; Waksman Institute of Microbiology, Rutgers University, Piscataway, NJ 08901-8520, USA; Department of Biology, Central Michigan University, Mount Pleasant, MI 48859, USA; School of Plant Sciences, University of Arizona, Tucson, AZ 85721, USA; Department of Statistics, Iowa State University, Ames, IA 50011, USA; School of Plant Sciences, University of Arizona, Tucson, AZ 85721, USA; Department of Biology, Central Michigan University, Mount Pleasant, MI 48859, USA; Waksman Institute of Microbiology, Rutgers University, Piscataway, NJ 08901-8520, USA; Department of Plant Biology, Rutgers University, New Brunswick, NJ; Genetics, Development and Cell Biology Department, Iowa State University, Ames, IA 50011, USA; Department of Agronomy, Iowa State University, Ames, IA 50011, USA

## Abstract

NAKED ENDOSPERM1 (NKD1), NKD2, and OPAQUE2 (O2) are transcription factors important for cell patterning and nutrient storage in maize (*Zea mays*) endosperm. To study the complex regulatory interrelationships among these 3 factors in coregulating gene networks, we developed a set of *nkd1, nkd2*, and *o2* homozygous lines, including all combinations of mutant and wild-type genes. Among the 8 genotypes tested, we observed diverse phenotypes and gene interactions affecting cell patterning, starch content, and storage proteins. From ∼8 to ∼16 d after pollination, maize endosperm undergoes a transition from cellular development to nutrient accumulation for grain filling. Gene network analysis showed that NKD1, NKD2, and O2 dynamically regulate a hierarchical gene network during this period, directing cellular development early and then transitioning to constrain cellular development while promoting the biosynthesis and storage of starch, proteins, and lipids. Genetic interactions regulating this network are also dynamic. The assay for transposase-accessible chromatin using sequencing (ATAC-seq) showed that O2 influences the global regulatory landscape, decreasing NKD1 and NKD2 target site accessibility, while NKD1 and NKD2 increase O2 target site accessibility. In summary, interactions of NKD1, NKD2, and O2 dynamically affect the hierarchical gene network and regulatory landscape during the transition from cellular development to grain filling in maize endosperm.

## Teaser

Three maize transcription factors interact and regulate a dynamic gene network that inhibits cellular development and promotes storage compound accumulation at grain filling.

**IN A NUTSHELL**

**Background:** The endosperm of cereal plants such as rice (*Oryza sativa*) and maize (*Zea mays*) serves as a storage tissue for nutrients, supplying energy for germination and the initial growth of seedlings. It is also a source for human food, livestock feed, and industrial applications. The endosperm develops in 2 stages, a cellular development phase characterized by extensive cell division and cell differentiation, and a second phase, grain filling, in which these cells accumulate storage materials, particularly starches and proteins. Three key transcription factors, NAKED ENDOSPERM1 (NKD1), NKD2, and OPAQUE2 (O2), play important roles in maize endosperm cellular development and storage material accumulation. The mutants of these factors showed several common defects in endosperm grain filling, and this suggests that they may have some related functions. However, we know little about how the 3 factors interactively regulate downstream genes and the regulatory landscapes that modulate endosperm development.

**Question:** How do NKD1, NKD2, and O2 coregulate gene networks and the regulatory landscape associated with endosperm development and how do they interact to control seed phenotype?

**Findings:** We developed a set of homozygous lines consisting of *nkd1, nkd2*, and *o2* mutants in all single, double, and triple mutant combinations, as well as wild-type. We identified synthetic phenotypes suggesting that the 3 factors interactively affect endosperm development. We found that the 3 factors may constrain hormone responses, cell wall organization, as well as other cellular developmental processes, and promote starch metabolism, lipid storage, and storage protein accumulation during the transition from cellular development to storage compound accumulation. These processes are regulated through a dynamic, hierarchical gene network, and the 3 factors function as the central regulators. We also reported potential direct target genes of NKD1 and NKD2 along with their chromatin accessibility status and differentially expressed genes between wild-type and each mutant.

**Next steps:** In the future, we plan to test some key direct targets of NKD1 and NKD2 by developing mutant lines to further explore how they work together to regulate endosperm development.

## Introduction

Cereal endosperm is a nutrient storage tissue that provides energy for germination and early seedling development and is important for human food, livestock feed, and industrial commodity. Following double fertilization, the triploid endosperm undergoes coenocytic development (∼1 to 3 d after pollination, DAP) and cellularization (∼3 to 4 DAP) before entering the proliferation and differentiation stage (starting at ∼4 DAP), where cells differentiate into various cell types with specific locations, cellular characteristics, and biological functions (Olsen 2004; Sabelli and Larkins 2009b; Leroux et al. 2014).

In maize (*Zea mays*), major cell types include starchy endosperm (SE), basal endosperm transfer layer (BETL), aleurone (AL), and embryo surrounding region (ESR), which become morphologically identifiable at around 4 to 5 DAP, followed by further differentiation of other cell types, such as subaleurone (SA) and conducting zone (CZ) (Leroux et al. 2014). In SE, mitotic activity peaks around 8 DAP, after which the cells undergo a transition to endoreduplication (Sabelli and Larkins 2009a; Sabelli and Larkins 2009b). Soon after, SE enters grain filling where cells accumulate starch and storage proteins, resulting in a massive increase of kernel weight (Sabelli and Larkins 2009a, 2009b; Dante et al. 2014). AL is a single, outermost layer of endosperm that plays an important role during germination to digest starch and storage proteins in SE for seedling growth (Hoecker et al. 1999; Becraft and Yi 2011). In contrast to SE, after ∼10 DAP, AL cells form thickened walls, accumulate protein and lipid but not starch, and do not undergo endoreduplication nor programed cell death (Burton and Fincher 2014). BETL contains multiple layers of transfer cells located at the basal region of endosperm adjacent to the maternal placento–chalazal region. The cells feature cell wall ingrowths that increase the surface area of cell membrane to facilitate transport of nutrient compounds into the endosperm (Zheng and Wang 2010). ESR cells are small, densely cytoplasmic cells surrounding the embryo at early stages and are believed to function in communication between the embryo and endosperm (Schel et al. 1984; Cosségal et al. 2007; Leroux et al. 2014).

From ∼8 to ∼16 DAP is an important transition from cellular development to nutrient accumulation for grain filling. Among the many factors involved in regulating these processes, NAKED ENDOSPERM1 (NKD1), NKD2, and OPAQUE2 (O2) are central. NKD1 and NKD2 are duplicate INDETERMINATE DOMAIN (IDD) transcription factors (TFs) encoded by *nkd1* (Zm00001d002654) and *nkd2* (Zm00001d026113), while O2 is a BASIC LEUCINE ZIPPER (bZIP) domain TF encoded by *o2* (Zm00001d018971) (Schmidt et al. 1987, 1990; Yi et al. 2015; Jiao et al. 2017).

Double mutants of *nkd1 nkd2* have pleiotropic effects on endosperm development. They disrupt AL cell patterning and cause multiple layers of peripheral cells with compromised cell identity, indicating that NKD1 and NKD2 function to limit the number of AL cell layers and promote AL cell differentiation (Yi et al. 2015). Additionally, NKD1 and NKD2 are important factors in grain filling. In SE, they positively regulate storage protein genes, and the *nkd1 nkd2* double mutant showed reduction in total starch abundance, altered starch branching patterns, and irregular starch granules (Gontarek et al. 2016).

Mutations in *o2* cause increased endosperm lysine content and opaque kernel phenotype due to reduction of storage protein content (Mertz et al. 1964; Hartings et al. 1989; Schmidt et al. 1992). O2 binds to a GENERAL CONTROL OF NITROGEN4-like motif (or O2-box) at promoters of target genes (Schmidt et al. 1990, 1992). Chromatin immunoprecipitation sequencing (ChIP-seq) analysis showed that O2 direct target genes are mainly involved in nutrient reservoir activity (genes encoding zein storage proteins) (Li et al. 2015; Zhan et al. 2018). In addition, O2 forms protein complexes with other TFs, such as PROLAMIN-BOX BINDING FACTOR1, OPAQUE2 HETERODIMERIZING PROTEIN1 (OHP1) and OHP2, ZmbZIP22, and ZmMADS47, to coordinately regulate zein production, starch biosynthesis, and nutrient metabolism (Dai et al. 2021). Additionally, O2 is involved in AL and SE cell fate specification and differentiation, abiotic response, and endoreduplication (Kowles and Phillips 1985; Zhan et al. 2018). These studies suggest that O2 is a key regulator involved in multiple gene networks and metabolic pathways during grain filling.

NKD1, NKD2, and O2 have close relationships among one another. The *nkd1* or *nkd2* single mutants showed increased expression of *nkd2* or *nkd1*, respectively (Yi et al. 2015), and a dual-luciferase reporter assay showed that NKD2 may directly repress the expression of *nkd1* (Gontarek et al. 2016). This feedback regulation may help limit NKD TF levels. In addition, NKD1 and NKD2 can form homodimers or heterodimers (Gontarek et al. 2016), although the biological significance of this is unknown. NKD1 and NKD2 can also transactivate expression of the *o2* gene and in *nkd1 nkd2* double mutants, and *o2* is downregulated (Gontarek et al. 2016). O2 also transactivates *nkd2*, and O2 and NKD2 may coactivate *floury2*, *bzip17*, and *tryptophan aminotransferase related3* genes, which are involved in storage protein biosynthesis (Zhan et al. 2018). Further, there is a strong overlap of differentially expressed genes (DEGs) between the *nkd1 nkd2* double mutant and *o2* mutant and also of putative target genes between NKD2 and O2 (Gontarek et al. 2016; Zhan et al. 2018).

In this study, we sought to explore how NKD1, NKD2, and O2 coregulate gene networks associated with endosperm development and how they interact to control seed phenotype. A set of homozygous lines was developed consisting of *nkd1, nkd2*, and *o2* mutants in all combinations of single, double, and triple mutants as well as wild-type (WT). They manifested diverse kernel phenotypes that suggested that NKD1, NKD2, and O2 interactively affect endosperm development. RNAseq was performed on developing endosperm tissues from 8 (cellular development, early onset of storage product accumulation) to 16 DAP (extensive storage compound accumulation). We constructed a hierarchical regulatory network through weighted gene coexpression network analysis (WGCNA) and NKD1 and NKD2 DNA affinity purification sequencing (DAP-seq) analysis, as well as assay for transposase-accessible chromatin using sequencing (ATAC-seq) analysis. We found NKD1, NKD2, and O2 function dynamically and interactively to regulate gene networks that play important roles in the grain-filling transition, to constrain the cell proliferation and differentiation processes and promote nutrient biosynthesis and storage.

## Results

### Genetic interactions among *nkd1*, *nkd2*, and *o2* genes during grain filling

**Figure 1.**
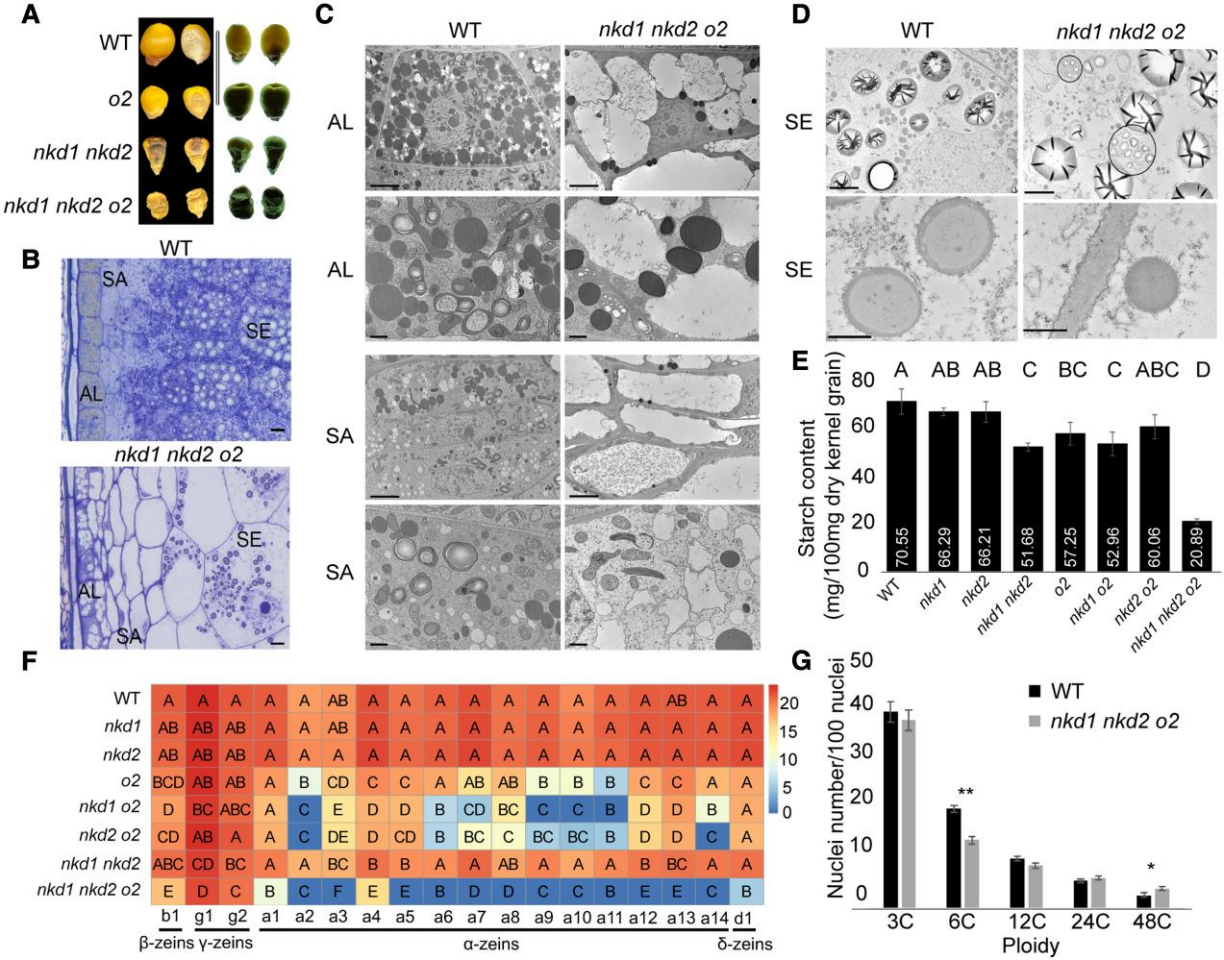
The defective phenotypes of the *nkd1 nkd2 o2* triple mutant. A**)** Kernel phenotypes of WT, *o2*, *nkd1 nkd2*, and *nkd1 nkd2 o2*. Black background: kernel surface view; white background: kernel opaqueness. Scale bar = 10 mm. **B)** Light micrographs of WT and *nkd1 nkd2 o2*. Scale bar = 10 *μ*m. **C)** TEM images of AL and SA in WT and *nkd1 nkd2 o2*. Rows 1 and 3, low magnification. Scale bars = 5 *μ*m. Rows 2 and 4, high magnification. Scale bars 1 *μ*m. **D)** TEM images of starch (Row 1) and protein bodies (Row 2) in SE. Scale bar = 5 *μ*m (Row 1) and 0.5 *μ*m (Row 2). Aggregated starch granules are encircled. **E)** Starch content among the 8 genotypes. Values are the means of 4 biological replicates from 4 independent ears of each genotype, each replicate a pool of 3 endosperms. The letters above the bars represent significance levels (nonoverlapping letters indicate significant differences at *P* < 0.05) by pairwise *t*-test. Error bars represent Ses. **F)** Zein profiles among the 8 genotypes. Numbers following the lower case letters at the bottom of each column (a1, a2, …, a14) represent different HPLC peaks indicating different isoforms of the corresponding types of zeins. The letters in the heatmap grid represent significance levels (*P* < 0.05) by pairwise *t*-test. The colors in the heatmap grid represent relative amount of corresponding zein isoforms in arbitrary units (A.U.) based on the means of 3 biological replicates from 3 independent ears of each genotype, each replicate a pool of 3 endosperms. **G)** Relative nuclei number of the corresponding ploidy between WT and *nkd1 nkd2 o2*. Values are means of 4 biological replicates, from 2 independent ears of each genotype, 2 replicates per ear, each replicate a pool of 3 endosperms. * *P* < 0.05 and **P < 0.01, respectively, by pairwise *t*-test. Error bars represent Sd.

To investigate combinatory effects of *nkd1*, *nkd2* and *o2* genes, homozygous mutant combinations were developed in a W22 inbred background. The *o2* mutants have normal size kernels with reduced translucency (Mertz et al. 1964), and the *nkd1 nkd2* double mutant shows small, wrinkled kernels with reduced translucency (Yi et al. 2015; Gontarek et al. 2016), whereas the *nkd1 nkd2 o2* triple mutant manifested a highly shrunken and wrinkled phenotype (Fig. 1A; Supplemental Fig. S1A). In addition, *nkd1 o2* double mutant kernels have an indentation below the crown region not seen in any other genotypes (Supplemental Fig. S1, A to C and Table S1). These phenotypes suggest that the *nkd1, nkd2*, and *o2* genes interact during grain development.

Light and transmission electron microscopy (TEM) were used to study the endosperm phenotypes at the cellular level, and various phenotypic alternations were observed in AL, SA, and SE (Fig. 1, B to D; Supplemental Figs. S2 to S4). Compared with WT, *nkd1 nkd2* and the *nkd1 nkd2 o2* triple mutant have multiple layers of poorly differentiated AL cells containing multiple large vacuoles, fewer lipid bodies, and irregular cell wall orientation (Fig. 1C; Supplemental Fig. S2). WT has 1 to 2 layers of SA cells containing partial features of AL, such as lipid bodies, and partial features of SE, such as starch grains and small developing protein bodies (Fig. 1C), whereas *nkd1 nkd2* and *nkd1 nkd2 o2* have 2 to 5 layers of highly vacuolated SA cells lacking protein bodies (Fig. 1C). In the triple mutant SE, starch granules were much fewer than WT (Fig. 1D; Supplemental Figs. S2 to S4). Interestingly, aggregate starch granules were present in all samples of the triple mutant endosperm. We measured total starch content in mature dry kernels, and compared with WT, the starch contents were significantly decreased in *o2*, *nkd1 nkd2* (Hasjim et al. 2009; Gontarek et al. 2016), and *nkd1 o2* mutants, whereas the triple mutant content was less than 30% of WT and was significantly lower than all other genotypes (Fig. 1E).

While TEM of WT protein bodies shows a core of light-staining α- and δ-zeins surrounded by dark-staining β- and γ-zeins (Lending and Larkins 1989), the protein bodies of *o2* single mutant showed light spots randomly distributed in dark-staining background (Supplemental Fig. S4A). Protein bodies of other mutants showed variable sizes and internal staining (Supplemental Fig. S4). HPLC zein profiling of 16 DAP endosperms of all genotypes containing *o2* mutants showed a significant decrease of most α-zein isoforms (Fig. 1F). The *nkd1 nkd2* double mutants showed greater dark-staining areas with reduced, variably sized light staining areas, compared with WT (Supplemental Fig. S4A), and zein profiling showed a significant decrease in levels of 3 α-zein isoforms (a4, a5, and a12) (Fig. 1F). Protein body morphology of the triple mutant is similar to *nkd1 o2*, but the zein profiles show that levels of most isoforms in all classes of zeins are significantly lower than any other genotype (Fig. 1, D and F; Supplemental Fig. S4A). These data indicate that the *nkd1* and *nkd2* mutants enhance the effect of *o2* on storage protein accumulation.

To test the influence of NKD1, NKD2, and O2 on endoreduplication, nuclei from 16 DAP endosperm of all 8 genotypes were FACS-sorted by ploidy. The *o2* mutant increased ploidy levels in endosperm cells compared with WT (Supplemental Fig. S5F; Kowles and Phillips 1985). The *nkd1* mutant, alone or in combination with *nkd2*, *o2*, or both, tended to decrease the proportion of lower ploidy nuclei, particularly 6C, and increase the proportion of nuclei 24C and 48C (Fig. 1G; Supplemental Fig. S5, A, C, and D). These data suggest that NKD1, NKD2, and O2 function to constrain endoreduplication.

### The *nkd1, nkd2*, and *o2* genes function in the transition from cellular development to nutrient accumulation

**Figure 2.**
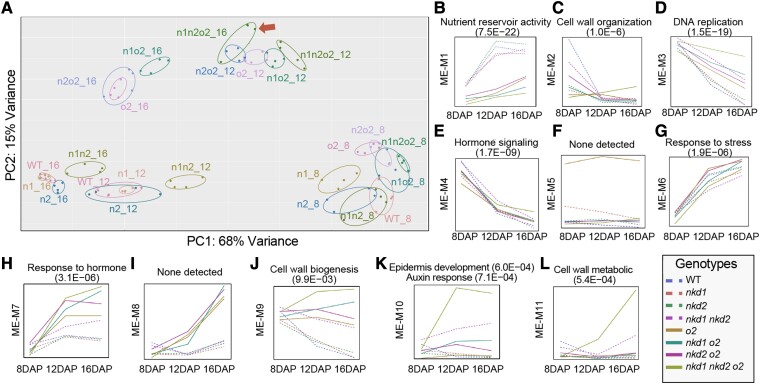
Transcriptomic relationships between genotypes from 8 to 16 DAP. **A)** PCA of the 8 genotypes at 8, 12, and 16 DAP in 4 biological replicates (n1 = *nkd1*, n2 = *nkd2*, n1o2_12 represents *nkd1 o2* double mutant at 12 DAP). The arrow highlights the transcriptome of the *nkd1 nkd2 o2* triple mutant at 16 DAP, which is close to the *o2* single/double/triple mutants at 12 DAP. **B**) to **L**). Temporal change of the eigengenes (MEs) of the 8 genotypes at 3 timepoints within each of the 11 coexpression modules (M1 to M11). Enriched gene ontology (GO) terms are shown for each respective module with the FDR in parentheses.

To investigate the regulatory relationship among the 3 TFs, a transcriptomic analysis was performed on endosperm tissue from each genotype at 8, 12, and 16 DAP. Among all 96 samples (8 genotypes × 3 timepoints × 4 biological replicates), an average of 48,924,625 reads per sample were aligned to B73 reference genome (Zm-B73-REFERENCE-GRAMENE-4.0) (Jiao et al. 2017) with a median alignment rate of 84.2%. Principal component analysis (PCA) of the 96 transcriptomes showed major effects due to developmental time, with secondary effects due to genotype (Fig. 2A). Eight DAP transcriptomes were well separated from later samples indicating a dramatic change of transcriptomes between 8 and 12 DAP, likely reflecting the major change in activity as the endosperm transitions from early cell proliferation and differentiation to grain-filling stages.

Genotype had a greater impact at 12 or 16 DAP than at 8 DAP. Strikingly, genotypes containing mutant *o2* genes were distinct from *O2*+genotypes (containing a homozygous WT *O2* allele, regardless of the genotypes at other loci) at 12 and 16 DAP reflecting the key function of O2 in grain filling. Double mutant *nkd1 nkd2* genotypes consistently separated from single mutant and *Nkd1+ Nkd2+* (WT *Nkd1* and *Nkd2* alleles) as expected due to their redundancy. Triple mutant *nkd1 nkd2 o2* transcriptomes appeared to cluster with earlier timepoints, possibly reflecting a developmental retardation of the triple mutant endosperm.

To study the effects of these TFs on gene expression patterns, gene readcounts of each mutant were compared against WT at each timepoint using DESeq2 (Love et al. 2014). DEGs were called when log2-fold change (log2FC) was >1 or <−1 and false discovery rate (FDR) <0.05. A total of 14,099 genes were differentially expressed in at least one mutant genotype for at least one timepoint. At 8, 12, and 16 DAP, a total of 6,235, 8,406, and 10,510 DEGs, respectively, were called from at least one mutant genotype, indicating an increasing impact of the 3 TFs on the transcriptome over time (Supplemental Fig. S6 and Data Set 1).

To investigate the gene expression network in which the 3 key TFs participate, WGCNA (Langfelder and Horvath 2008) was performed using log2-transformed value of transcripts per million reads (TPM) of the 14,099 DEGs. A total of 12,458 genes were assigned into 11 coexpression modules (M1 to M11) correlated with *nkd1, 2*, and/or *o2* expression levels. The sizes of these modules range from 100 (M5) to 3,501 (M4) genes (Supplemental Table S2 and Data Set 2). Six of the 11 modules correlated with 2 or more of the TFs suggesting substantial overlap in the genes and processes they regulate. GO enrichment analysis was performed to study the biological processes associated with each module. Every module, except M5 and M8, was enriched (FDR < 0.05) for several GO terms, and the top 1 or 2 informative and significantly enriched terms are shown in Fig. 2 and Supplemental Data Set 3. Cell wall development and hormone pathways are notably prevalent.

To explore the temporal dynamics of this network, we analyzed the behavior of each genotype over time. Module eigengenes (MEs) are defined as the first principal component of the expression matrix of the corresponding module and reflect a general expression level of the genes in that module (Langfelder and Horvath 2007). Eigengene values were calculated for each genotype at each timepoint, within every module (Fig. 2, B to L). In general, most mutants behaved similar to one another within most modules. A notable exception is the *nkd1 nkd2 o2* triple mutant, which increased over time relative to other genotypes in modules M2, M9, M10, and M11 (Fig. 2, C, J, K, and L). Intriguingly, these modules are involved in cell wall and epidermal development, suggesting that as development progresses, the 3 TFs may cooperate to restrict cellular development.

### NKD1, NKD2, and O2 coregulate different sets of coexpression modules at each timepoint

The coexpression network above was generated using all DEGs across DAPs (pooled timepoint WGCNA). MEs of most modules did not show substantial separation among genotypes at 8 DAP (Fig. 2), likely because later timepoints had more DEGs which skewed the analysis. Also, gene expression is dynamic, so we hypothesize that coexpression patterns may change over time. To test this hypothesis and to unveil expression network information that may have been missed by pooled timepoint WGCNA, we performed WGCNA using DEGs of each DAP individually (individual timepoint WGCNA).

**Figure 3.**
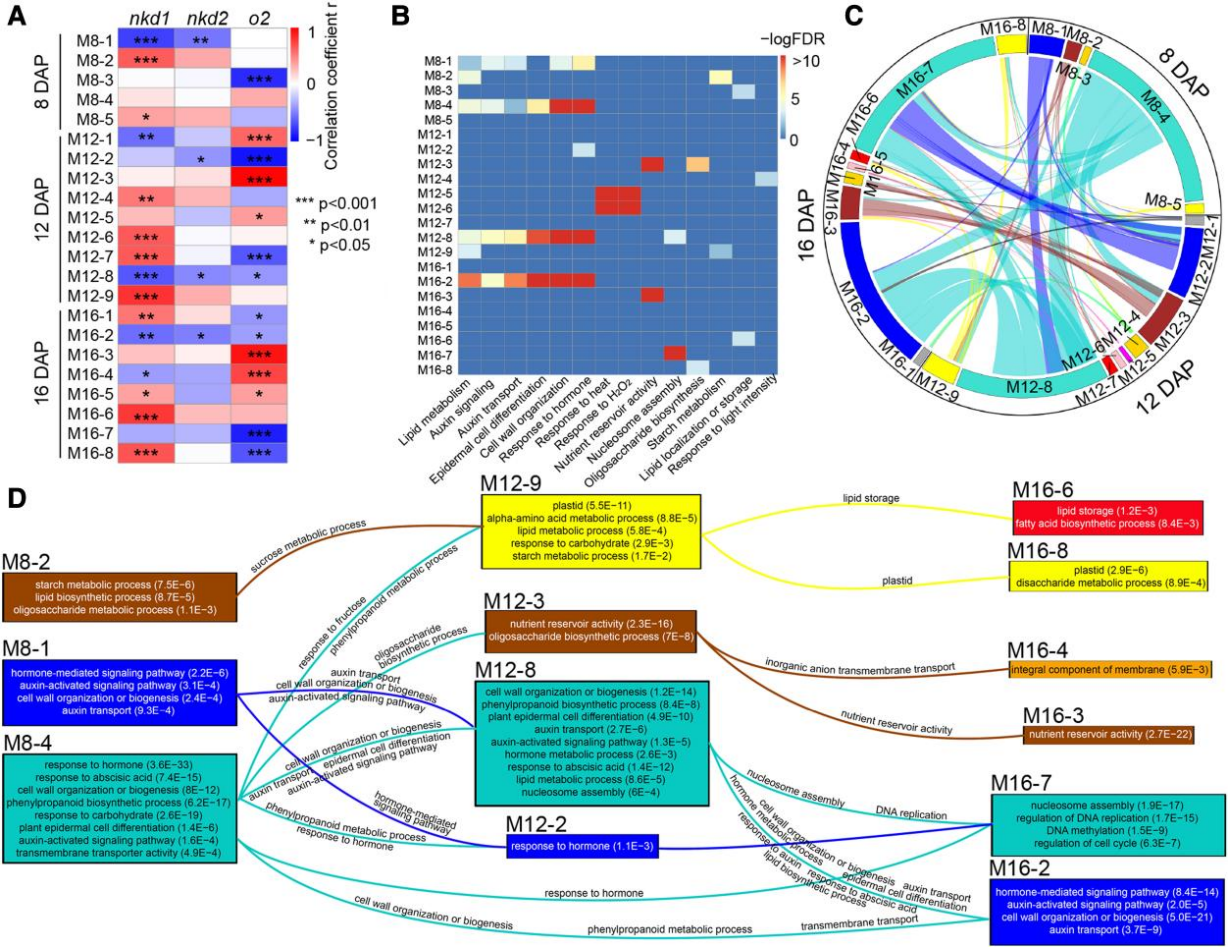
Coexpression modules correlated with *nkd1, 2*, and *o2* at individual timepoints and dynamic relationships of modules between different timepoints. **A)** The correlation of *nkd1*, *nkd2*, and *o2* gene expression with the ME of corresponding coexpression modules at different DAPs (e.g. M12-5 represents the Module #5 at 12 DAP). The heatmap indicates positive (upregulation) or negative (downregulation) correlations between the 3 key TFs and the corresponding modules. *P < 0.05, **P < 0.01, and ***P < 0.001, respectively, by Pearson correlation test. **B)** Enriched gene ontology (GO) terms of the corresponding coexpression modules. The heatmap indicates –logFDR of the GO terms. **C)** The chord diagram shows the dynamic change of coexpression modules over time. The size of the arcs is proportional to the number of genes in the corresponding modules. The chord connecting 2 arcs represents genes expressed at both timepoints in the corresponding modules. **D)** The split and convergence of the modules over time. The main enriched GO terms are listed in the boxes corresponding to the modules. The FDR of each GO term is shown in parentheses. Lines connecting boxes represent genes overlapping between the 2 modules. The GO term(s) of the overlapping genes are listed above or below the line.

There were 5, 9, and 8 coexpression modules identified at 8, 12, and 16 DAP, respectively, and each module correlates with at least 1 of the 3 TFs (Fig. 3A). The size of modules varied across timepoints, ranging from 148 (M8-3) to 3855 (M8-4) genes, from 96 (M12-5) to 3112 (M12-8) genes, and from 132 (M16-5) to 3676 genes (M16-7) at 8, 12, and 16 DAP, respectively (Supplemental Data Sets 4 to 6). As anticipated, modules were identified at 8 DAP that were not called in pooled timepoint WGCNA, which supports the hypothesis of dynamic coexpression networks. These contained additional functional information; e.g. M8-1, negatively correlated with *nkd1* and *nkd2*, may regulate auxin signaling, auxin transport, and lipid metabolism, as well as cell wall organization. M8-2, positively correlated with *nkd1*, may regulate starch and lipid metabolism (Fig. 3, A and B; Supplemental Data Set 7).

Modules at 12 and 16 DAP were generally similar to those called by pooled timepoint and had many similarities to each other in TF correlations and functions. For instance, both M12-8 and M16-2 are negatively correlated with *nkd1, 2*, and *o2* and are associated with auxin signaling, cell wall organization, and plant epidermal cells; both M12-3 and M16-3 are positively correlated with *o2*, and both associated with nutrient reservoir activity (Supplemental Data Set 7). These patterns are consistent with many modules called by pooled timepoint WGCNA (such as M1, M7, M9, and M10) (Fig. 2, B, H, J and K); however, some modules were identified specific to 12 DAP, such as M12-4, 5, and 6 associated with stress response (Fig. 3B).

Several modules at different timepoints had similar TF correlations and enriched GO terms; hence, we traced module gene members over time to reveal the dynamic relationships among modules. The result showed that modules split and converge from one timepoint to the next (Fig. 3, C and D). For example, 12.9% and 25.9% of genes in M12-8 were shared with M8-1 and M8-4, respectively, 47.1% and 14.3% of genes in M16-2 were shared with M12-8 and M8-4, respectively, and 22.4% and 19.8% of genes in M16-7 were shared with M12-2 and M12-8, respectively. The similarities in gene membership reflect functional similarities among modules at different timepoints as seen in enriched GO term similarities of these modules (Fig. 3, B and D). For example, genes involved in sucrose metabolic processes in M8-2 and fructose response in M8-4 merge to M12-9 and may contribute to carbohydrate response and starch metabolic processes, whereas genes involved in lipid storage and other processes in plastids in M12-9 split into 2 modules at 16 DAP associated with more specific functions (Fig. 3D).

### NKD1, 2, and O2 interact to regulate gene networks

Coexpression network analysis suggested that NKD1, NKD2, and O2 may interact to regulate gene networks; MEs of double or triple mutants showed different dynamic patterns compared with corresponding single mutants (Fig. 2), and some modules are correlated with more than 1 of the 3 key TFs (Fig. 3A). To further analyze the interactions of NKD1, 2, and O2 in regulating gene networks, a multifactorial analysis was performed on DEGs of each timepoint. If the log2FC value of a DEG in mutant TF1, given WT TF2, is significantly different from mutant TF1, given mutant TF2, this gene is considered to be affected by the interaction between TF1 and TF2.

**Figure 4.**
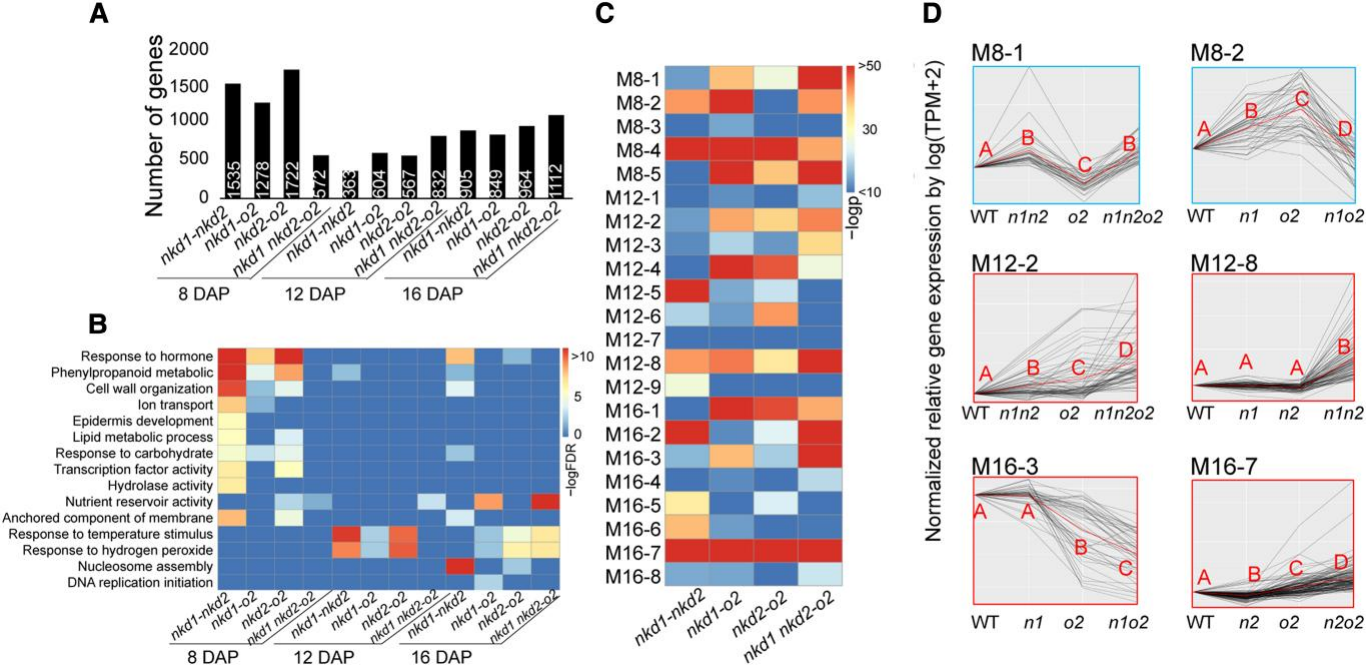
The effects of interaction between *nkd1, nkd2*, and *o2* on DEGs and coexpression networks. **A)** Number of DEGs affected by corresponding interactions (e.g. *nkd1-o2* represents the interaction effect of *nkd1* and *o2*). **B)** Gene ontology (GO) terms affected by corresponding interactions (e.g. *nkd1-o2* represents the interaction effect of *nkd1* and *o2*). The heatmap represents –logFDR as shown on the scale (blue = 0, red = 10 or greater). **C)** Coexpression modules affected by corresponding interactions. The heatmap indicates the significance level, via hypergeometric test, of the overlap between the genes affected by corresponding interactions and the genes in the corresponding modules (e.g. M8-1 = module 8-1). Colors represent –log*p* as shown on the scale. **D)** The normalized relative gene expression by genotypes in selected modules. The uppercase letters represent significance levels (*P* < 0.05) by pairwise *t*-test of mean gene expression values of each genotype. The upper 2 graphs show overall suppression, while the lower 4 illustrate enhancement interactions between 2 individual factors. For example, D (M16-3) showed that the genes in this module were influenced by enhancement effect of *nkd1–o2* interaction (expression in *nkd1 o2* is significantly lower than the additive effect of *nkd1* and *o2*). TPM, transcripts per million; M, module.

Interactions were identified among all TF combinations, and the number of DEGs affected varied over time (Fig. 4A; Supplemental Data Set 8). NKD1–NKD2, NKD1–O2, and NKD2–O2 interactions affect greater numbers of genes at 8 DAP than 12 or 16 DAP, whereas the interaction among NKD1–NKD2–O2 influences gradually increasing numbers of genes from 8 to 16 DAP (Fig. 4A). The regulatory functions of each interaction are dynamic. For instance, the NKD1–NKD2 interaction most strongly impacts genes in hormone response, phenylpropanoid metabolism, and cell wall formation at 8 DAP, response to temperature and hydrogen peroxide at 12 DAP, and nucleosome assembly at 16 DAP (Fig. 4B). The effect of NKD1–NKD2–O2 interaction on nutrient reservoir activity gradually becomes stronger from 8 to 16 DAP and also affects response to temperature and hydrogen peroxide at 16 DAP (Fig. 4B).

Interactions among the 3 key TFs were also dynamic in regulating coexpression modules. Hypergeometric tests between interaction-affected DEGs and coexpression modules showed that M8-1, 2, 4, 5; M12-4, 5, 8; and M16-1, 2, 3, 7 have significant overlap (−log*p* > 50) with at least one of the interaction pairs (Fig. 4C), indicating that these modules are likely to be influenced by interactions among the TFs. Interactions caused diverse effects on gene expression in different modules (Fig. 4D). For instance, genes in M8-1 were downregulated in *o2* mutant compared with *nkd1 nkd2* double mutant, whereas there was no significant difference between *nkd1 nkd2 o2* triple mutant and *nkd1 nkd2* (Fig. 4D). Hence, mutations of *nkd1* and *nkd2* may suppress the effect of mutation of *o2* in this module. In genes of M16-3, no significant expression change was identified between WT and *nkd1*, whereas gene expression decreased more in *nkd1 o2* double mutant than *o2* single mutant (Fig. 4D), suggesting that *nkd1* may enhance the effect of *o2* in this module.

### Functions of NKD1, 2, and O2 gene networks in endosperm tissue types

**Figure 5.**
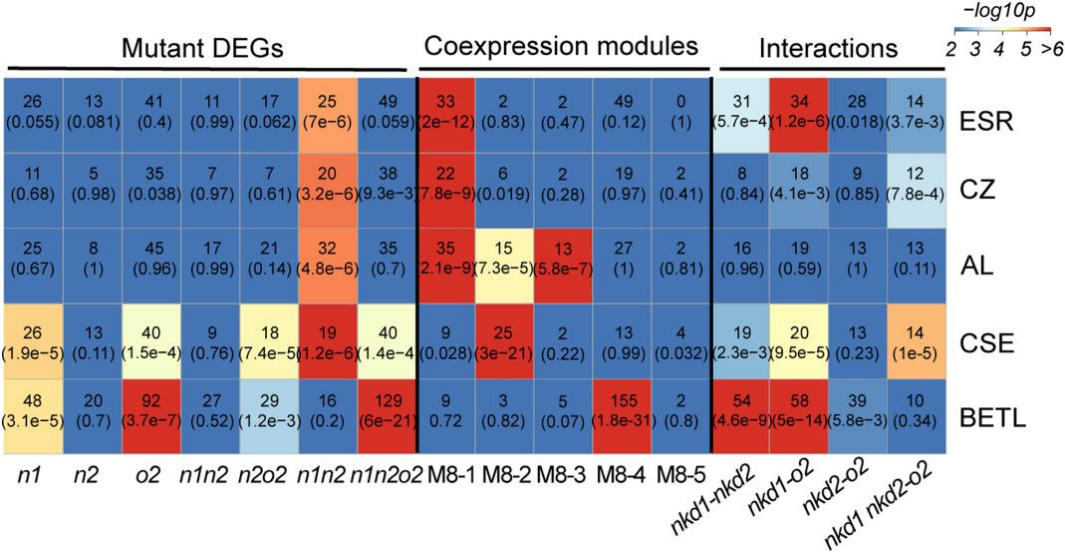
Overlap of endosperm tissue-preferential genes with DEGs, coexpression modules, or genes affected by interactions at 8 DAP. The upper number in the grid represents the number of overlapping genes, and the number in the parentheses represents the corresponding *P*-value via hypergeometric test. *n1, n2, o2, n1o2, n2o2, n1n2,* and *n1n2o2* represent DEGs, relative to WT, of *nkd1, nkd2, o2, nkd1 o2, nkd2 o2, nkd1 nkd2,* and *nkd1 nkd2 o2.* M8-1 to M8-5 represent coexpression modules 8-1 to 8-5. *nkd1-nkd2, nkd1-o2, nkd2-o2*, and *nkd1 nkd2-o2* represent interaction effects of *nkd1* with *nkd2*, *nkd1* with *o2*, *nkd2* with *o2,* and *nkd1 nkd2* with *o2*, respectively. CSE, central SE.

To explore how NKD1, NKD2, and O2 regulate gene expression in specific endosperm tissues, data from this study were compared with previously published genes expressed preferentially in specific tissue types, which were obtained by performing RNAseq on specific tissues isolated from 8 DAP endosperm using laser capture microscopy (Zhan et al. 2015). Hypergeometric tests were performed to test overlap between these tissue-specific endosperm genes and 8 DAP genes from our study that were: (i) differentially expressed in each mutant genotype, (ii) correlated with coexpression modules, or (iii) affected by interactions of the 3 key TFs. DEGs of *nkd1 nkd2* have significant overlap with genes specific to every cell type except BETL, whereas *o2* DEGs are only associated with BETL and CSE (Fig. 5). Coexpression modules M8-1 and M8-3 contain significant numbers of AL genes (Fig. 5). These modules are involved with lipids, hormones, and cell wall (Fig. 3B), functions of particular importance to AL. M8-1 is also enriched for CZ and ESR genes, while M8-2 (starch metabolism) overlaps with CSE genes. Various gene interactions affected genes expressed in every tissue except AL.

### NKD1, 2, and O2 regulate coexpression modules directly or indirectly through hub TFs

**Figure 6.**
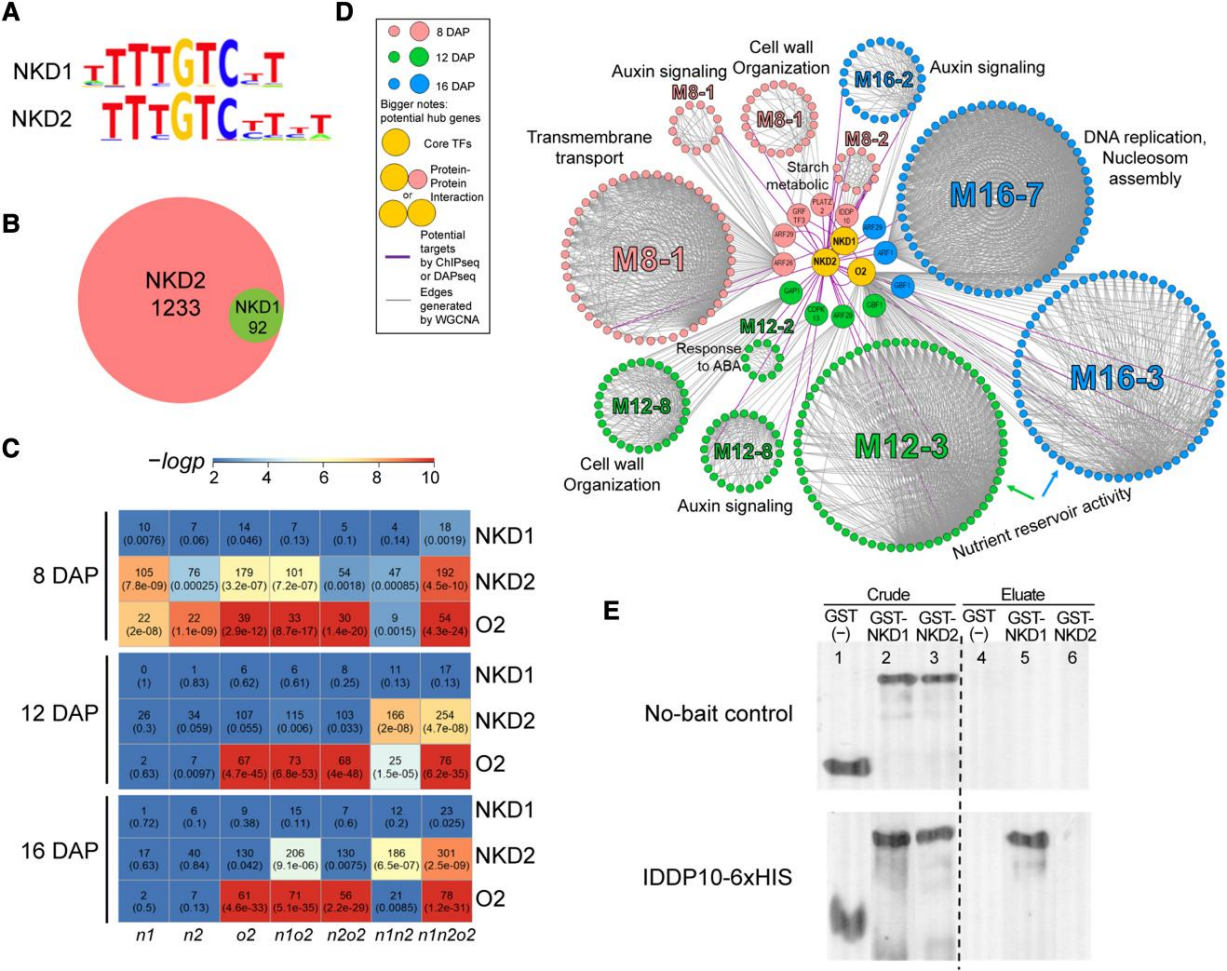
DAP-seq analysis of NKD1 and NKD2 and hierarchical network layout based on individual timepoint WGCNA and DAP-seq. **A)** Binding motif of NKD1 and NKD2. **B)** Venn diagram illustrating the number of NKD1 and NKD2 bound genes. **C)** Hypergeometric test of the NKD1, NKD2, and O2 bound genes overlapping with DEGs of the mutant genotypes at 8, 12, and 16 DAP. The upper number in the grid represents the number of overlapping genes, and the number in parentheses represents the corresponding *P*-value. **D)** Hierarchical gene network layout of selected coexpression modules generated via Cytoscape 3.7.2. The size of the circles is based on the tiers of the genes in the hierarchy. The heavy edges connect the TFs and their potential direct targets, and the light edges connect coexpressed genes in the corresponding modules that are not direct targets. **E)** Pull-down assay showed protein–protein interaction between IDDP10 and NKD1. IDDp10-6xHIS was used as bait, and NKD1 or NKD2 fused with GLUTATHIONE-S-TRANSFERASE (GST) were prey. In vitro translated proteins were mixed and applied to a 6xHIS affinity resin, followed by elution. Prey proteins were detected by immunoblot analysis using anti-GST antibodies. Lanes 1 to 3 show crude input, 4 to 6 show eluate. The IDDp10-6xHIS bait was able to bind to GST-NKD1 (lane 5, lower panel) but not to GST-NKD2 (lane 6), nor GST alone (lane 4). In the absence of bait protein, no prey proteins were retained by the resin (upper panel).

To further characterize regulatory functions, DNA affinity purification and sequencing (DAP-seq) was performed on NKD1 and NKD2 proteins produced in vitro. Motif enrichment analysis showed potential binding motifs of NKD1 and NKD2 similar to reported (Gontarek et al. 2016) with a TTTGTC core (Fig. 6A). There were 1,951 peaks called for NKD1 and 56,855 for NKD2, of which 93 and 1,692 peaks were located within 3 kb of annotated transcription start sites (TSSs) of 92 and 1,233 genes, respectively (Fig. 6B; Supplemental Data Sets 9 and 10). All NKD1 bound genes were contained among the NKD2 bound genes. The NKD2 consensus binding sequence, TTTGTC[CT]T, was found at 225,434 sites throughout the genome, of which 13.96% were located within 3 Kb of the annotated TSS. Among the bound sites, 28.76% were within 3 Kb of the annotated TSS for NKD1, and 21.82% for NKD2 (Supplemental Fig. S7, A to C). Among the sites flanking the annotated TSS, bound sites had a propensity to occur in 2 or more copies per gene, whereas the unbound sites mainly occurred as singlets (Supplemental Fig. S7, D and E).

The NKD1 and NKD2 bound genes, together with published ChIP-seq O2 targets (Zhan et al. 2018), were incorporated with DEGs identified for mutant genotypes at each timepoint. The intersection between DAP-seq targets or published ChIP-seq O2 targets and DEGs were considered potential direct targets of NKD1, 2, or O2 regulation. O2 ChIP-seq targets had significant (*P* < 1e−6) overlap with *o2* mutant DEGs across all 3 timepoints (Fig. 6C). NKD2 DAP-seq targets significantly overlapped (*P* < 1e−6) with DEGs of *nkd1*, *o2*, *nkd1 o2*, and *nkd1 nkd2 o2* at 8 DAP, whereas at 12 and 16 DAP, only *nkd1 nkd2* and *nkd1 nkd2 o2* DEGs overlapped (Fig. 6C). NKD1 DAP-seq targets had low overlap due to the low number of peaks. Among the putative direct targets of NKD1 and NKD2, 25% to 50% were TFs, and the most significantly enriched GO term was “transcription factor activity” (GO:0003700) across all timepoints (Table 1; Supplemental Table S3).

**Table 1.** GO terms of NKD1 or NKD2 potential direct targets at 8, 12, and 16 DAP

TF	DAP	GO	Description	FDR
NKD1	8	GO:0003700	Transcription factor activity, sequence-specific DNA binding	4.30E−02
NKD1	8	GO:0046983	Protein dimerization activity	4.30E−02
NKD2	8	GO:0003700	Transcription factor activity, sequence-specific DNA binding	1.80E−28
NKD2	8	GO:0046983	Protein dimerization activity	6.60E−13
NKD2	8	GO:0007389	Pattern specification process	6.30E−16
NKD2	8	GO:0009888	Tissue development	1.10E−08
NKD2	8	GO:0009755	Hormone-mediated signaling pathway	1.10E−08
NKD2	8	GO:0001708	Cell fate specification	1.50E−05
NKD2	8	GO:0019252	Starch biosynthetic process	7.10E−04
NKD1	12	GO:0044255	Cellular lipid metabolic process	2.50E−02
NKD2	12	GO:0003700	Transcription factor activity, sequence-specific DNA binding	7.50E−24
NKD2	12	GO:0046983	Protein dimerization activity	2.30E−05
NKD2	12	GO:0045735	Nutrient reservoir activity	1.40E−03
NKD2	12	GO:0009755	Hormone-mediated signaling pathway	3.00E−12
NKD2	12	GO:0048316	Seed development	7.30E−07
NKD2	12	GO:0008610	Lipid biosynthetic process	7.70E−06
NKD2	12	GO:0005984	Disaccharide metabolic process	1.20E−04
NKD1	16	GO:0044249	Cellular biosynthetic process	4.40E−02
NKD2	16	GO:0003700	Transcription factor activity, sequence-specific DNA binding	1.70E−29
NKD2	16	GO:0046983	Protein dimerization activity	1.80E−14
NKD2	16	GO:0045735	Nutrient reservoir activity	5.20E−03
NKD2	16	GO:0009755	Hormone-mediated signaling pathway	5.00E−11
NKD2	16	GO:0048316	Seed development	1.40E−07
NKD2	16	GO:0009311	Oligosaccharide metabolic process	2.10E−04
NKD2	16	GO:0034728	Nucleosome organization	2.20E−04
NKD2	16	GO:0008610	Lipid biosynthetic process	6.30E−03

To further investigate how O2, NKD1, and NKD2 coregulate gene expression, hub genes were called with module membership scores greater than 0.8, indicating significant connectivity within the module. Hub genes that were potential targets of NKD1 and NKD2 included genes encoding TFs, kinases, and protein processing-related factors (Table 2), suggesting that NKD1 and 2 may regulate coexpression modules in a hierarchical pattern. O2 target hub genes included the TF genes *bzip17* and *g-box binding factor1* (*gbf1*) indicating that O2 also functions hierarchically within this network. Genes for AUXIN RESPONSE FACTOR1 (ARF1) and ARF29 were among the potential NKD2 targets, and since published DAP-seq targets were available for these factors (Galli et al. 2018; Zhan et al. 2018), they were also incorporated to build a gene regulatory network with selected modules (Fig. 6D). At 8 DAP, NKD1 and NKD2 regulate M8-1 responsible for cell wall organization, auxin signaling, and transmembrane transport possibly through TFs PLANT AT-RICH SEQUENCE AND ZINC BINDING PROTEIN2 (PLATZ2), GROWTH REGULATING FACTOR TF (GRFTF3), and ARF29.

**Table 2.** Potential NKD1, NKD2, and O2 modulated hub genes of selected modules

Module	DAP	Hub gene id	Hub gene product	Description	Module membership score	Potential target of
M8-1	8	Zm00001d029437	PLATZ2	PLATZ transcription factor family protein	0.938873691	NKD1, NKD2
M8-1	8	Zm00001d021362	GRFTF3	Homolog of growth-regulating factor 9	0.864321005	NKD1, NKD2
M8-1	8	Zm00001d017908	BHLH156	Homolog of transcription factor BIM3	0.846721007	NKD2
M8-1	8	Zm00001d026540	ARF29	Homolog of auxin response factor 5	0.800065026	NKD2
M8-2	8	Zm00001d007382	BHLH96	Homolog of Transcription factor ICE1	0.824640415	NKD2
M12-2	12	Zm00001d031522	ARF1	Auxin response factor 1	0.975753404	NKD2
M12-2	12	Zm00001d024488	MYB155	Homolog of MYB domain protein 118	0.959466374	NKD2
M12-2	12	Zm00001d051568	TUBTF6	Tubby-like F-box protein 6	0.952357799	NKD2
M12-2	12	Zm00001d036416	CDPK13	Calcium-dependent protein kinase13	0.879817661	NKD2
M12-3	12	Zm00001d018971	O2	Regulatory protein opaque-2	0.976845488	NKD2
M12-3	12	Zm00001d046937	BZIP17	Basic leucine zipper 17	0.978356856	O2
M12-3	12	Zm00001d039065	GBF1	G-box binding factor 1	0.947779763	O2
M12-3	12	Zm00001d038288	MYBR13	Putative MYB DNA-binding domain superfamily protein	0.963581808	NKD2
M12-5	12	Zm00001d033987	HSFTF17	Homolog of heat stress transcription factor A-6b	0.907691436	NKD2
M12-8	12	Zm00001d021362	GRFTF3	Homolog of growth-regulating factor 9	0.903802679	NKD1, NKD2
M12-8	12	Zm00001d029437	PLATZ2	PLATZ transcription factor family protein	0.89337778	NKD1, NKD2
M12-8	12	Zm00001d033174	GAP1	Golgi associated protein homolog	0.93285082	NKD2
M12-8	12	Zm00001d017726	GLK11	Homeodomain-like superfamily protein	0.912192373	NKD2
M12-8	12	Zm00001d007173	ABI39	Homolog of B3 domain-containing transcription repressor VAL2	0.899143178	NKD2
M12-8	12	Zm00001d026540	ARF29	Homolog of auxin response factor 5	0.837692812	NKD2
M16-2	16	Zm00001d017900	DOF42	Homolog of Dof zinc finger protein DOF5.4	0.943253299	NKD1, NKD2
M16-2	16	Zm00001d008808	MYBR24	Putative MYB DNA-binding domain superfamily protein	0.940741423	NKD1, NKD2
M16-2	16	Zm00001d002234	HB75	Homeodomain leucine zipper family IV protein	0.935349746	NKD2
M16-2	16	Zm00001d020670	HB11	Homolog of homeobox-leucine zipper protein HAT3	0.92812669	NKD2
M16-3	16	Zm00001d018971	O2	Regulatory protein opaque-2	0.923257678	NKD2
M16-3	16	Zm00001d039065	GBF1	G-box binding factor 1	0.872074634	O2
M16-3	16	Zm00001d046937	BZIP17	Basic leucine zipper 17	0.830433051	O2
M16-7	16	Zm00001d031522	ARF1	Auxin response factor 1	0.988098645	NKD2
M16-7	16	Zm00001d024488	MYB155	Homolog of MYB domain protein 118	0.963596519	NKD2

While not identified as a potential target of NKD1 or NKD2, *idd protein10* (*iddp10*) was called as a hub gene for M8-2, associated with starch metabolism. IDDP10 is closely related to NKD1 and 2 (Yi et al. 2015; Coelho et al. 2018) and can heterodimerize with NKD1 based on a pull-down assay (Fig. 6E), indicating NKD1 and IDDP10 may coregulate M8-2. At 12 DAP, NKD2 potential targets *cyclin dependent kinase13* (*cdpk13*) and *myb155* are hub genes for M12-2 that contribute to ABA response; ARF29 and GOLGI ASSOCIATED PROTEIN1 (GAP1) may regulate M12-8 associated with auxin signaling and cell wall organization, respectively. At 16 DAP, ARF1, a potential target of NKD2, may regulate M16-7 responsible for DNA replication and nucleosome assembly. Furthermore, as a direct target of NKD2, O2 itself is a hub gene regulating M12-3 and M16-3, involved in nutrient storage (Fig. 6D).

To further explore the architecture of the gene networks, DAP-seq was performed on select downstream TFs from key modules (Supplemental Fig. S8A). For example, GBF1 is a TF in modules M12-3 and M16-3 and is a direct target of O2 (Zhan et al. 2018). GO enrichment analysis of its DAP-seq targets suggested that GBF1 contributes to amino acid and protein production in both modules (Supplemental Fig. S8, A and B and Data Set 11). In M12-9, *heat shock factor TF10* (*hsftf10*) is a potential direct target of NKD2 () and shares targets with NKD2, including *chalcone flavone isomerase1* (*chi1*), *chloroplast rna splicing protein2* (*crs2*), and *oil yellow1* (*oy1*), functioning in the plastid (Supplemental Fig. S8, A and C and Data Set 12).

### NKD1, NKD2, and O2 interactively affect endosperm chromatin accessibility

TF action is often apparent by alterations in chromatin accessibility (Xie and Liu 2019). To investigate how interactions among NKD1, NKD2, and O2 affect the endosperm regulatome, we performed ATAC-seq for 16 DAP endosperms of all 8 genotypes (2 biological replicates per genotype). Among 16 sequenced samples, 20,478 to 49,640 (mean: 34,766) peaks were called, representing potential regions of accessible chromatin. Distribution of the peaks on chromatin varies among samples: from 54.35% to 66.73% of peaks are located at distal intergenic regions (>3 kb upstream and >300 bp downstream of an annotated gene), while 6.7% to 12.1% are located in promoter regions (within 3 kb flanking an annotated TSS), and 4.62% to 8.88% are located within 1 kb flanking an annotated TSS (Supplemental Fig. S9 and Data Sets 13 to 20).

**Figure 7.**
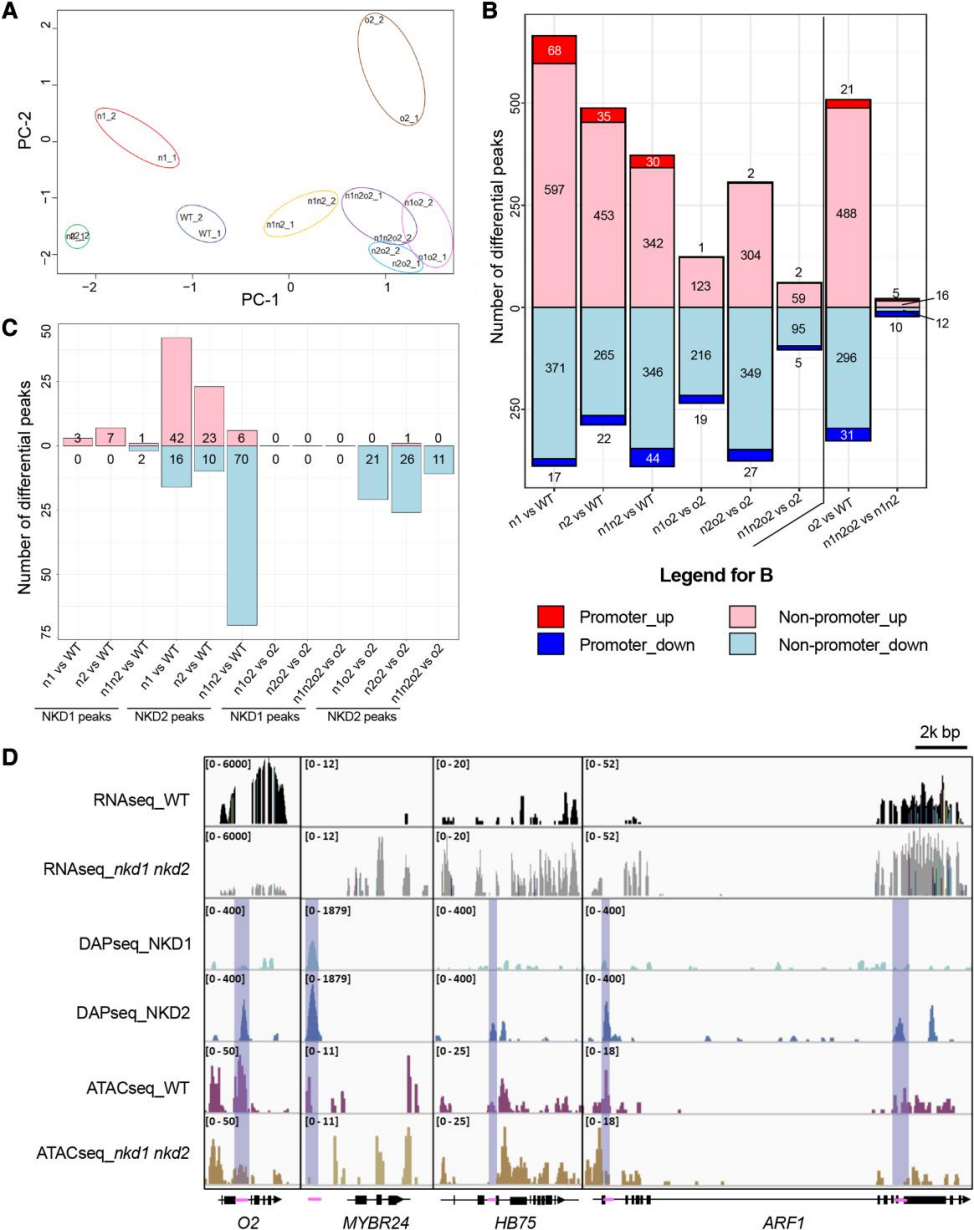
Chromatin accessibility profiles among genotypes of 16 DAP maize endosperm. **A)** PCA of accessible peaks among 8 genotypes (in 2 biological replicates). **B)** Number of differential peaks categorized by more opening (up) or closing (down) peaks at promoter or nonpromoter regions. **C)** Number of overlapping peaks between the ATAC-seq and DAP-seq analysis. **D)** Tracks of RNAseq (WT and *nkd1 nkd2*), DAPseq (NKD1, NKD2), and ATAC-seq (WT and *nkd1 nkd2*) of selected potential direct targets of NKD1 and/or NKD2 listed at Table 2. Differential peaks are highlighted with shading, and the potential NKD1 and/or NKD2 binding regions represented with magenta bars on the gene models. Numbers in brackets represent scale range of corresponding tracks. O2 is downregulated in *nkd1 nkd2* (log2FC = −1.20, adjusted *P* = 0.0022), and MYBR24, HB75, and ARF1 are upregulated in *nkd1 nkd2* (log2FC = 2.75, 1.75 and 1.87; adjusted *P* = 0.017, 1.41E−11, and 1.70E−14, respectively).

PCA showed that the regulatome of WT, *nkd1*, *nkd2*, *o2* single mutants and *nkd1 nkd2* double mutant are well separated from one another, whereas *o2* double or triple mutants are clustered together (Fig. 7A). This suggests that NKD1, NKD2, and O2 interactively influence the endosperm regulatory landscape. To further explore this possibility, differential peak/accessibility analysis of *nkd1*, *nkd2*, and *nkd1 nkd2* versus WT was compared with *nkd1 o2*, *nkd2 o2*, and *nkd1 nkd2 o2* versus *o2*. Compared with WT, *nkd1* and *nkd2* single mutants have overall greater numbers of open regions (upregulated peaks) than closed regions (downregulated peaks), whereas *nkd1 nkd2* double mutant has similar numbers of open and closed regions (Fig. 7B). In contrast, in *o2*-backgrounds, all *nkd1*, *nkd2*, and *nkd1 nkd2* mutants have greater numbers of closed than open regions, suggesting that O2 may influence the ways NKD1 and NKD2 interact with chromatin. On the other hand, we also compared the differential peak/accessibility of *o2* versus WT with *nkd1 nkd2 o2* versus *nkd1 nkd2.* In the WT (or *Nkd1^+^ Nkd2*^+^) and *nkd1 nkd2* mutant backgrounds, *o2* has 836 and 43 differential peaks, respectively (Fig. 7B). This difference indicates that NKD1 and NKD2 also influence how O2 interacts with chromatin.

To investigate chromatin regions that may be directly regulated by NKD1 or NKD2, DAP-seq data were compared with the differential peaks from ATAC seq, and peaks that overlapped between the 2 datasets were identified. Fewer overlapping peaks were identified for *nkd* mutants in an *o2* mutant background than *O2^+^* (Fig. 7C), which agrees with the above finding that O2 affects chromatin accessibility of NKD1 and NKD2. Interestingly, in the *O2^+^* background, the number of open chromatin regions for NKD1 or NKD2 are greater in single mutants of *nkd2* or *nkd1*, respectively, whereas when both *nkd1* and *nkd2* are mutated; there is a sharp increase of closed chromatin regions (Fig. 7C).

To examine how the differential chromatin accessibility could affect the expression of NKD1 or NKD2 potential target genes, we incorporated the tracks of WT and *nkd1 nkd2* RNAseq and ATAC-seq, as well as NKD1 and 2 DAPseq peaks for selected hub genes from coexpression modules at 16 DAP (Fig. 7D; Table 2). For the *o2* gene, RNAseq showed decreased readcounts in the *nkd1 nkd2* mutant compared with WT. In intron 2 of *o2*, DAPseq identified a NKD2 binding site that is coincident with a region of chromatin that ATAC-seq showed had decreased accessibility in the *nkd1 nkd2* mutant (Fig. 7D). This suggests that NKD2 may upregulate *o2* by binding to the second intron. Similarly, there is a differentially accessible NKD2 binding site in intron 2 of *homeobox TF75* (*hb75*), which shows elevated expression in *nkd1 nkd2* mutant, suggesting NKD2 binding may downregulate *hb75* (Fig. 7D). NKD1 and NKD2 may downregulate *mybr24* by binding to a promoter element (Fig. 7D), while multiple NKD2 binding sites in the transcribed regions of *arf1* may downregulate expression (Fig. 7D).

To elucidate the nature of increased accessibility regions, motif enrichment analysis was performed on potential NDK2 DAP-seq target regions overlapping with differential accessibility regions caused by *nkd1*, *nkd2*, or *nkd1 nkd2* mutants. The result showed that binding motifs of IDD family TFs are most significantly enriched, followed by TFs of other families, including HMG, bZIP, bHLH, DOF, RING-type, and MADS (Supplemental Table S4).

## Discussion

### Synthetic phenotypes of *nkd1*, *nkd2*, and *o2* mutants may be associated with disruption of grain filling processes

The *nkd1 o2* double mutant and *nkd1 nkd2 o2* triple mutant produced synthetic kernel phenotypes not observed in single or *nkd1 nkd2* double mutants (Fig. 1; Supplemental Fig. S1). While the *nkd1* single mutant did not produce a visible phenotype, *nkd1 o2* double mutants have wrinkled kernels in addition to the opaque phenotype of *o2*. Similarly, the *nkd2 o2* double mutant showed only the opaque phenotype of *o2*, while *nkd1 nkd2 o2* showed extremely small, wrinkled kernels. These genetic interactions indicate that NKD1, NKD2, and O2 have related functions in kernel development. Wrinkled kernel phenotypes often result from disruption of grain filling processes (Tsai and Nelson 1966; James et al. 1995; Zhang et al. 2016, 2019a, 2019b; Yang et al. 2021). Protein bodies and starch granules normally form a matrix that produces the hard, vitreous endosperm and desirable physiochemical properties (Wu et al. 2010; Holding 2014). Defects in either proteins or starch may produce a loose unstable matrix that results in opaque, chalky, or shrunken endosperm (Wu et al. 2010). This is consistent with our phenotypic and transcriptomic analyses. Mutant combinations with wrinkled kernels caused decreased starch accumulation and defects in starch grain morphology (Fig. 1, D and E). They also caused smaller protein bodies and pronounced deficiencies in zein storage protein accumulation (Fig. 1, D and F; Supplemental Figs. S2 to S4). WGCNA indicated that regulating the accumulation of grain storage products is a major function of these 3 TFs. At 8 DAP, *nkd1* is positively correlated with module M8-2 associated with starch metabolism (Fig. 3, A, B, and D), and at 16 DAP, *nkd2* and *o2* interact to regulate M16-3 associated with storage protein production (Fig. 4, B to D).

### NKD1 and NKD2 paralogs have different interaction patterns with O2

As described, *Nkd1* and *Nkd2* genes mutually regulate each other in a feedback mechanism that partially compensates single mutants of either gene (Yi et al. 2015; Gontarek et al. 2016). The ATAC-seq analysis gave further support to this mutual compensation model. The single mutant of *nkd1* has a greater number of opened chromatin regions for NKD2, while *nkd2* enhances chromatin accessibility for NKD1 (Fig. 7C), whereas this is not observed in the *nkd1 nkd2* double mutant. Increased open chromatin regions in *nkd1* may enhance binding by NKD2 and vice versa, to further contribute to the genetic complementation for each gene by the other.

Although clearly paralogs, this study showed that NKD1 and NKD2 have divergent functions; they are correlated with different sets of coexpression modules (Fig. 3A) and have different interaction patterns with O2 (Fig. 4). The *nkd2 o2* double mutant has a similar kernel phenotype as *o2*, whereas *nkd1 o2* showed a wrinkled kernel phenotype (Supplemental Fig. S1). In addition, single mutants of either gene result in over a thousand DEGs, especially at 8 DAP (Supplemental Fig. S6). Thus, NKD1 and NKD2 have divergent functions in addition to redundancy. Two possible models include: (i) neofunctionalization, when one or both duplicated genes may gain new functions and (ii) subfunctionalization, when one or both duplicated genes may partially lose the function of their ancestral gene (Pickett and Meeks-Wagner 1995; Moore and Purugganan 2005).

### NKD1, 2, and O2 affect each other's regulatory landscapes

We assayed the influence of these TFs on chromatin by comparing mutant with WT using ATAC-seq. Interestingly, the effects of NKD1 and/or NKD2 on chromatin accessibility were greater in the presence of a WT *O2^+^* allele than if *o2* was mutant. Further, in an *O2^+^* background, *nkd1* and *nkd2* mutants showed increased numbers of accessible sites, compared with *Nkd1^+^* or *Nkd2^+^*, whereas in an *o2* mutant background, *nkd1* and *nkd2* mutants showed decreased numbers of accessible sites (Fig. 7). Notably, fewer potential direct target sites of NKD1 and NKD2 were available in *o2* mutant than in *O2^+^* WT. This indicates that O2 plays an important role in modeling the global NKD1 and NKD2 regulatory landscape. Conversely, the number of differentially accessible chromatin peaks of *o2* mutant versus *O2^+^* WT is nearly 20 times greater in the *Nkd1^+^ Nkd2*^+^ background than in the *nkd1 nkd2* mutant background, indicating that NKDs also affect the O2 regulatory landscape.

The mode by which NKDs and O2 impact the regulatomes is unclear, but several scenarios can be envisaged. O2 directly promotes expression of the *nkd2* gene; NKD2 negatively regulates expression of the *nkd1* gene, while NKD1 and NKD2 positively regulate the expression of the *o2* gene (Gontarek et al. 2016; Zhan et al. 2018). This dynamic regulatory circuit could contribute to variations in NKD1 and NKD2 activity between *O2*^+^ and *o2* backgrounds, and O2 activity between *Nkd1^+^ Nkd2*^+^ and *nkd1 nkd2* backgrounds. The regulatory landscapes of NKD1, NKD2, and O2 may also be affected indirectly due to increased endoreduplication in the *o2* and *nkd1 nkd2* mutant backgrounds, respectively (Supplemental Fig. S5) (Kowles and Phillips 1985; Sabelli 2012). Increased endoreduplication may cause increased chromatin condensation and disruption of global chromatin accessibility for TFs (van Zanten et al. 2011; Sabelli et al. 2013). Consistent with O2 functioning to restrict endoreduplication, we found that O2 was negatively correlated with coexpression module M16-7, responsible for nucleosome assembly and regulation of DNA replication (Fig. 3, A, B, and D). NKD1 and 2 may also genetically interact with O2 and coregulate the module M16-7 (Fig. 4, B to D). Furthermore, auxin has been implicated in promoting endoreduplication (Sabelli 2012), and *ARF1* was identified as a potential direct target of NKD2 and a hub gene of M16-7 (Figs. 6D and 7G; Table 2).

### NKD1, 2, and O2 function dynamically in the transition to grain filling

Beginning at about 8 DAP, maize endosperm undergoes a transition from lag phase, characterized by cell proliferation and differentiation, to grain filling with extensive storage compound accumulation (Wu et al. 2022). Our data indicate these 3 genes function dynamically throughout this transition. Mutants show defects in cell patterning and differentiation as well as impaired accumulation of starch, storage proteins, and lipids (Fig. 1; Supplemental Figs S2, S3, and S4) (Schmidt et al. 1992; Hasjim et al. 2009; Yi et al. 2015; Gontarek et al. 2016). WGCNA of WT aligned well with processes of endosperm development and the transition to grain filling, reflecting decreases in cell wall production-related processes and increases in nutrient reservoir activity from 8 to 16 DAP (Fig. 2), whereas various mutant combinations of *nkd1*, *nkd2*, and *o2* manifested different temporal patterns in these processes.

Strikingly, at later stages, the *nkd1 nkd2 o2* triple mutant had elevated cell wall production-related processes (Fig. 2, C, J, and L) and a dramatically delayed increase of nutrient accumulation (Fig. 2B), suggesting that the transition from lag phase to grain filling might be retarded. This was further supported by PCA showing that the triple mutant transcriptome at 16 DAP clustered with other *o2* mutant genotypes at 12 DAP (Fig. 2A). The genetic interactions between *nkd1*, *nkd2*, and *o2* also underwent a transition from cell wall organization, hormone response, and plant epidermis development at 8 DAP to nutrient reservoir activity, nucleosome assembly, and DNA replication at 16 DAP (Fig. 4B). In summary, these 3 TFs have key roles in both cellular development and grain filling, and they function and interact dynamically during the transition between these stages.

### Working model of NKD1, 2, and O2 regulating the transition processes during the endosperm development

This study demonstrated that O2, NKD1, and NKD2 are key regulators that interact in a dynamic network that performs changing functions over developmental time, from primarily cellular development at 8 DAP to storage compound accumulation at 16 DAP. The coexpression modules undergo migration of genes from 8 to 16 DAP, resulting in merged or split modules from one timepoint to another, as the network undergoes temporal changes in developmental functions (Fig. 3, C and D).

**Figure 8.**
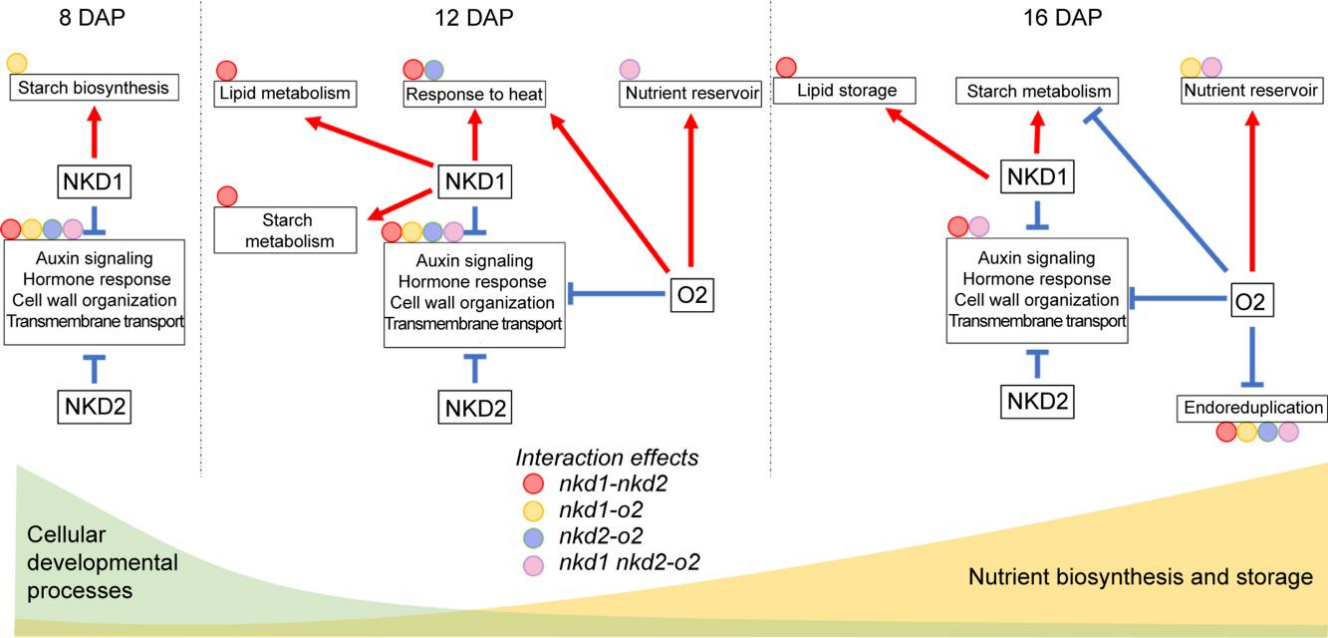
Models of NKD1, 2, and O2 regulating the transition processes in endosperm development. Processes are shown in boxes. Positive regulation is shown by red arrows, negative regulation by blue T-shaped lines, and interactions are represented by circles attached to the corresponding boxes.

Some of the key functions are summarized in Fig. 8. NKD1 and NKD2 function to restrict auxin signaling, cell wall organization, and other cellular developmental processes throughout endosperm development, which may help explain the mutant phenotype of extra cell layers (Yi et al. 2015). These functions are accomplished through modules M8-1, 12-8, and 16-2. Hub genes at 8 DAP include *ARF29*, *PLATZ2*, and *GRFTF3*, which are potential direct targets of NKD1, NKD2, or both. *PLATZ2* encodes a plant AT-rich sequence and zinc-binding (PLATZ) protein. In rice (*Oryza sativa*), a PLATZ factor, SHORT GRAIN6 (SG6), influences grain size by regulating multiple cellular processes, including cell cycle and cell differentiation (Zhou and Xue 2020). *GRFTF3* is a putative ortholog of Arabidopsis (*Arabidopsis thaliana*) *GROWTH-REGULATING FACTOR9* (*GRF9*) (Table 2), which regulates cell growth (Omidbakhshfard et al. 2018).

By 12 DAP, additional hub genes include *gap1*, *g2-like TF11* (*glk11*), and *aba insensitive39* (*abi39*) (Fig. 6D; Table 2). GAP1 is a putative ortholog of the Arabidopsis REVERSIBLY GLYCOSYLATED POLYPEPTIDE1 (RGP1), which is a Golgi-associated protein involved in the cell wall biosynthesis (Welner et al. 2017). GLK11 is homologous to Arabidopsis ALTERED PHLOEM DEVELOPMENT (APL), associated with asymmetric cell divisions and differentiation of phloem (Bonke et al. 2003). ABI39 is homologous to Arabidopsis VP1/ABI3-LIKE2 (VAL2), a TF that maintains the embryonic state by interacting with PRC2 to modify chromatin (Suzuki et al. 2007; Yuan et al. 2021). Beginning at 12 DAP, O2 also participates in negatively regulating developmental processes via M12-8 and M16-2.

Functions in storage compound accumulation have been well documented for all 3 TFs (Fig. 1) (Yi et al. 2015; Gontarek et al. 2016; Zhang et al. 2016; Zhan et al. 2018; Deng et al. 2020). At 8 DAP, the module M8-2 is associated with early starch biosynthesis, and NKD1 and NKD2 potentially regulate M8-2 by modulating or interacting with the hub genes *bhlh96* and *iddp10* (Figs. 3A, 3B, and 6D; Table 2). While not a target of either NKD1 or NKD2 regulation, IDDP10 can physically interact with NKD1 (Fig. 6E) and has also been proposed to interact with NKD1 in leaf mesophyll cells (Coelho et al. 2018). Additionally, the Arabidopsis AtIDD5 is a positive regulator of *STARCH SYNTHASE4* (*SS4*) and may control starch granule formation (Ingkasuwan et al. 2012).

From 8 to 16 DAP, the transition from cellular development to nutrient storage is associated with dynamic interactions of NKD1, 2, and O2, which constrain the cellular developmental processes, and promote biosynthesis and storage of starch, proteins, and lipids. By 12 DAP, synthesis and accumulation of storage compounds have greatly increased, and all 3 TFs interact to promote this activity. The effects of these interactions are apparent from the synthetic phenotypes of *nkd1 o2* and *nkd1 nkd2 o2* mutants with dramatic grain-filling deficiencies affecting both starch and protein accumulation (Fig. 1, A, D, E, and F; Supplemental Fig. S1). O2 positively regulates zein synthesis and accumulation via modules M12-3 and M16-3 (Figs. 3 and 6D), and NKD1 and NKD2 synergistically interact with O2 on these processes (Fig. 4, B to D). In addition, NKD1 and NKD2 interact to promote lipid metabolism via modules M12-9 and M16-6 (Figs. 3, 5C, 6B, and 6C). Finally, NKD1, 2, and O2 may interact to modulate endoreduplication via module M16-7 (Figs. 3, 4D, 5B, 5C, and 5D), leading to differential distributions of chromosomal ploidy among WT and mutants (Fig. 1G; Supplemental Fig. S5). These processes are briefly summarized in our working model illustrated in Fig. 8.

## Materials and methods

### Plant materials and growth conditions

All *nkd1*, *nkd2*, and *o2* homozygous single, double, and triple mutants, as well as the WT maize (*Z. mays*), were derived from a cross between the *nkd1-Ds nkd2-null* (Ahern et al. 2009; Yi et al. 2015) double mutant and *o2* single mutant, each in the W22 genetic background. Segregating lines were developed by self-pollinating the triple heterozygote (+/*nkd1* +/*nkd2* +/*o2*), from which homozygotes of each genotype were identified. All plant materials were collected from descendants of the same segregating ears grown in the greenhouse at Molecular Biology Building or in the field at Curtiss Research Farm, Iowa State University (Ames, IA). Materials may be requested from the Maize Cooperation Genetic Stock Center, Urbana, IL, USA.

### RNA-seq and data analysis

Total RNA of 8, 12, and 16 DAP endosperms of *nkd1*, *nkd2*, and *o2* homozygous single, double, and triple mutants, as well as the WT counterpart (4 biological replicates, from 4 independent ears of each genotype, each replicate contains a pool of 3 endosperms), were extracted following the published LiCl precipitation-based protocol (Li et al. 2014), except that GeneJET RNA cleanup and concentration kit (Thermo Fisher Scientific, Waltham, MA, USA) were used for DNaseI-treated RNA purification. RNA quality control was performed via AATI Fragment Analyzer (Agilent, Santa Clara, CA, USA) at the DNA Facility, Iowa State University. High-quality (RQN > 9.0) RNA samples were sent to the HSC core, University of Utah (Salt Lake City, UT) for library preparation with Illumina TruSeq RNA Library Prep Kit (Illumina, San Diego, CA, USA) and 150 bp paired end sequencing using NovaSeq 6000 with the S4 cell.

The raw read quality control was performed via FastQC (https://www.bioinformatics.babraham.ac.uk/projects/fastqc/). The raw reads were mapped to B73 RefGen_v4 (AGPv4) by HISAT2 (v2.1.0) (Kim et al. 2015; Jiao et al. 2017). The mapped transcripts in output files were sorted and assembled by SAMtools (v1.9) and StringTie (v1.3.6) (Pertea et al. 2016), respectively. Readcounts were calculated and analyzed by HTSeq (v0.11.1) and DEseq2 (v1.26.0) (Love et al. 2014), respectively. DEGs were identified using a generalized linear model-based approach with the cutoff criteria log2FC >1 or <−1 and FDR < 0.05.

Gene coexpression network analyses were performed via WGCNA (Langfelder and Horvath 2008) using the TPM (in log_2_-transformed values) generated from StringTie (Pertea et al. 2016) of all DEGs following the previously published procedures (Zhan et al. 2015). For the pooled timepoint WGCNA, soft-thresholding power was set to 10 to build a signed network with the scale-free topology. The merge threshold and minimum module size were set as 0.1 and 60, respectively. For the individual timepoint WGCNA, soft-thresholding power was set to 7, 9, and 7 for 8, 12, and 16 DAP, respectively. The merge threshold and minimum module size were set as 0.3 and 60, respectively. The module hub genes were called with the Module Membership (MM) score >0.8. The network was visualized by Cytoscape 3.7.2.

Transcriptomics data were analyzed to investigate effects of NKD1, NKD2, and O2 interactions on gene expression using generalized linear model analysis based on multifactorial experimental design via edgeR (v3.38.4). We included 2 factors (Gene A and Gene B) and their interaction in the generalized linear model, where each factor has 2 levels (WT and mutant). The interaction effect between 2 TFs GeneA and GeneB can be interpreted as the difference of gene expression fold change (FC) for downstream genes introduced by Gene A (mutant/WT) given Gene B mutant vs Gene B WT. We controlled FDR at 5% using the Benjamini–Hochberg (BH) procedure.

### DAP-seq

DAP-seq genomic DNA libraries were generated as described previously (Bartlett et al. 2017). Briefly, 5 *μ*g of phenol:chloroform:IAA extracted DNA from maize B73 14-d-old seedlings was diluted in EB (10 mm Tris-HCl pH 8.5) and sheared to 200 bp fragments using a Covaris S2 sonicator. DNA was purified using AmpureXP beads with a 2:1 bead to DNA ratio and then end-repaired using an End-It kit (Lucigen) according to the manufacturer's recommendations. Samples were cleaned using a Qiaquick PCR purification kit (Qiagen), A-tailed using Klenow (3′ to 5′ exo-), cleaned with Qiaquick and ligated overnight with a truncated Illumina Y-adapter. Final libraries were purified using AMPureXP beads with a 1:1 bead to DNA ratio and eluted in 30 *μ*L of EB. DNA concentration was determined using the Qubit HS kit.

The pENTR/SD/D-TOPO constructs of *GBF1* and *HSFTH10* ORF were ordered from TFome stock center (Burdo et al. 2014) (https://abrc.osu.edu/stocks/766964). The entry clone constructs of *NKD1* and *2* ORF were made by cloning of the coding sequences of *NKD1* and *NKD2* into the pDONR221 donor vector (Thermo Fisher Scientific, Waltham, MA, USA) through the BP reaction. The primers for *NKD1* and *NKD2* cloning are shown in Supplemental Table S5. ORF clones were recombined into the pIX-HALO vector (Bartlett et al. 2017) using LR Clonase II (Thermo Fisher Scientific, Waltham, MA, USA) and purified using a Qiagen miniprep kit. Protein expression was carried out using the TnT SP6 Wheat Germ Protein Expression System (Promega, Madison, WI, USA) using 1 *μ*g of plasmid DNA according to the manufacturer's recommendations. Samples were incubated for 2 h at 30 °C. Fifty microliters of expression reaction was then diluted in 50 *μ*L of wash buffer (1×PBS with 0.005% [v/v] NP40) containing 10 *μ*L of MagneHaloTag beads (Promega, Madison, WI, USA). Samples were rotated for 1 h at room temperature and then washed 3 times with wash buffer. Beads were suspended in 50 *μ*L wash buffer, and 1 *μ*g of B73 genomic DNA library in 50 *μ*L of EB was added. Samples were then rotated at room temperature for 1 h and washed 7 times with wash buffer. To elute DNA, 30 *μ*L of EB was added, and samples were heated at 98 °C for 10 min. DNA was transferred to a new tube prior to 19 cycles of PCR enrichment and barcoding (Bartlett et al. 2017). DNA was sequenced on a NextSeq500 with 75 bp SE reads for *GBF1* and *HSFTH10* and on Novaseq with 150 bp PE for *NKD1* and *NKD2*.

Mapping and peak calling of DAP-seq samples were performed as follows. DAP-seq reads were trimmed using Trimmomatic (Bolger et al. 2014) with the following parameters: ILLUMINACLIP:TruSeq3-SE:2:30:10 LEADING:3 TRAILING:3 SLIDINGWINDOW:4:15 MINLEN:50. Trimmed reads were mapped to the B73v4 reference genome using Bowtie2.3.3. Mapped reads were filtered for reads containing >MAPQ30 using SAMtools (SAMtools view -b -q 30) (Li et al. 2009). Peaks were called using GEM v2.5 (Guo et al. 2012) with default thresholds and the following parameters: –d Read_Distribution_default.txt –k_min 6 –k_max 20 –outNP. Background peaks were subtracted using a HALO-GST negative control sample and a list of blacklisted regions from Galli et al. (2018) with the B73v3 sites converted to B73v4 coordinates. Peak distribution was analyzed via ChIPseeker (1.36.0) using default settings (Yu et al. 2015).

### Endosperm nucleus extraction and ATAC-seq

Frozen 16 DAP endosperms of *nkd1, nkd2*, and *o2* single, double, and triple mutants, as well as the WT counterpart were collected. ATAC-seq analysis contained 2 biological replicates from 2 independent ears of each genotype, each replicate contained a pool of 3 endosperms. Endoreduplication analysis consisted of 4 biological replicates, from 2 independent ears of each genotype, 2 replicates per ear, each replicate contained a pool of three endosperms. Crude nuclei were extracted following the published protocol (Lu et al. 2017) with the following modifications: 0.2 g of endosperm were ground in liquid nitrogen followed by addition of 2 mL of prechilled lysis buffer (15 mm Tris-HCl pH7.5, 20 mm NaCl, 80 mm KCl, 0.5 mm spermine, 5 mm 2-ME, and 0.2% [v/v] TritonX-100) to resuspend the ground powder. Then, the resuspension was filtered 3 times through miracloth. The crude nuclei were stained with DAPI (from Millipore Sigma, St. Louis, MO, USA) (4 *μ*L DAPI per ∼500 *μ*L filtered resuspension) and sent to the Flow Cytometry Facility at Iowa State University (Ames, IA) for nuclear counting and sorting using BD FACSAria III flow cytometer (BD Biosciences; San Jose, CA, USA). The nuclei with ploidies of 3C, 6C, 12C, 24C, and 48C were counted for endoreduplication analysis, and at least 50,000 triploid nuclei (3C) were sorted for ATAC-Seq library construction. The library was constructed using the Active Motif ATAC-Seq Kit (Active Motif, Carlsbad, CA, USA) following the manufacturer's manual. The library was sequenced using NovaSeq 6000 SP flow cell sequencing platform (DNA Facility, Iowa State University, Ames, IA) at 2X50 bp paired-end in 50 million bp depth. The raw read quality control was performed via FastQC (https://www.bioinformatics.babraham.ac.uk/projects/fastqc/), followed by the adapter trimming via Trimmomatic (v0.32). The Trimmomatic parameters were set as ILLUMINACLIP:Adapter2.fasta:2:30:10 LEADING:3 TRAILING:3 CROP:36 SLIDINGWINDOW:4:15 MINLEN:30, where the Adapter2.fasta file contained 2 adapter sequences: (i) CTGTCTCTTATACACATCT and (ii) AGATGTGTATAAGAGACAG. The trimmed reads were mapped to B73 RefGen_v4 (AGPv4) (Jiao et al. 2017) via Bowtie2 (v 2.4.2) (Langmead and Salzberg 2012) using a “–very-sensitive” set-up. The mapped reads in the output files were sorted, assembled, and filtered by SAMtools (v1.9) using default parameters, followed by the peak calling via MACS2 (Feng et al. 2012) using the parameters “-f BAMPE -g 2e9 –keep-dup all –nomodel –cutoff-analysis -q 1e-02 -n merged.”

### Microscopy

Kernels of *nkd1, nkd2*, and *o2* single, double, and triple mutants, as well as the WT counterpart, were harvested at 16 DAP and fixed for microscopy in 4% (v/v) formaldehyde/2% (v/v) glutaraldehyde in phosphate buffer, pH 7, and stored at 4 °C until processing. Kernels from ears of 3 plants of each of the 8 genotypes were cut in medial longitudinal hand sections, and then an abgerminal piece containing AL, SA, and SE was collected. Samples were washed in 50 mm sodium phosphate buffer pH 7 and then postfixed with buffered 2% osmium tetroxide at 4 °C overnight or 2 h at room temperature. After buffer washes, the kernels were dehydrated by ethanol series and embedded in Spurr's resin. Blocks were sectioned at 1 *µ*m using a RMC Powertome (Boekeler Instruments, Tucson, AZ, USA), stained with 1% (w/v) Toluidine Blue O in 1% sodium borate, and viewed and imaged with a Zeiss Axiophot equipped with a Zeiss AxioCam MRC (Carl Zeiss, Oberkochen, Germany). Thin-sections were cut at 70 to 80 *µ*m with a RMC Powertome, stained with Uranyless followed by lead citrate (both Electron Microscopy Sciences, Hatfield, PA, USA), and imaged with Hitachi HT7700 Scanning/Transmission Electron Microscope (Hitachi Instruments, Tokyo, Japan).

For protein body (PB) measurements, the diameter of PBs in electron micrographs at 1,000× magnification was measured with the line tool using Image Pro Premier software (Media Cybernetics, Rockville, MD, USA). Images of SE cells 7 or more cells internal from the pericarp were chosen, and the largest protein bodies in each image measured to avoid tangentially sectioned PBs. Three plants were analyzed per genotype, 3 to 7 images were analyzed per plant, with a range of 4 to 25 protein bodies measured per image, and a minimum sample size *n* = 171 per genotype.

### Starch content analysis and zein profiling

Total starch from mature kernels of *nkd1, 2*, and *o2* single, double, and triple mutants, as well as the WT counterpart (4 biological replicates from 4 independent ears of each genotype, each replicate contained a pool of 3 endosperms), were extracted following the published method (Dinges et al. 2001) with the following modifications: the endosperm starch was washed with chilled deionized water twice followed by chilled 80% ethanol (centrifuged 3,000 × *g* at 4 °C for 10 min after each liquid addition). The pellet was dissolved in 100% DMSO and boiled in a water bath for 1 h. The starch content was measured following the published procedures (Gontarek et al. 2016).

Zein proteins from 16 DAP endosperm of *nkd1, nkd2*, and *o2* single, double, and triple mutants, as well as WT (3 biological replicates from 3 individual ears of each genotype, each replicate contained a pool of 3 endosperms), were extracted based on published procedures (Wilson 1991) with the following modifications. The frozen endosperms were ground into powder in liquid nitrogen. One hundred grams of the power was suspended in 1 mL of the extraction buffer (70% ethanol, 61 mm sodium acetate, and fresh 5% [v/v] 2-mercaptoethanol), followed by incubation with shaking (225 rpm at 37 °C) for 1 h and centrifugation at 15,870 g for 10 min. The supernatant was sent to the Protein Facility at Iowa State University (Ames, IA) for HPLC.

### Pull-down assay

The pull-down assay was conducted using in vitro translated GST-tagged NKD1 and NKD2 proteins and 6X-Histidine (HIS)-tagged IDDP10 protein. The GST-tagged *NKD1* and *NKD2* entry constructs were previously made in the lab following published procedures (Gontarek et al. 2016). The 6X-HIS-tagged IDDP10 entry constructs were made by cloning the coding sequence of *iddp10* into pET34b vectors (Novogene Co. Durham, NC, USA), through the In-Fusion cloning approach following the manufacturer's manual (Takara Bio, Kusatsu, Shiga, Japan) using the primers shown in Supplemental Table S5. Then, the GST-tagged NKD1 and NKD2, and 6X-HIS-tagged IDDP10 fragments were In-Fusion (Takara Bio, Kusatsu, Shiga, Japan) cloned into the expression vector pF3K WG (BYDV) Flexi (Promega, Madison, WI, USA) using the primers shown in Supplemental Table S5. The pF3K expression constructs were in vitro transcribed and translated via the TnT SP6 High-Yield Wheat Germ Protein Expression System following the manufacturer's instructions (Promega, Madison, WI, USA). The GST-tagged NKD1 and NKD2 proteins or GST-only proteins (negative control) were mixed with 6X-Histidine (HIS)-tagged IDDP10 protein in phosphate buffered saline with 0.15 mm phenylmethylsulfonyl fluoride (PMSF) and incubated for 1 h at room temperature. The mixture was then passed through HIS-Select HF Nickel Affinity Gel resin (Sigma-Aldrich, St. Louis, MO, USA), and washed by 6X-HIS wash buffer (50 mm phosphate buffer pH 7.0, 300 mm NaCl, and 1 mm imidazole), followed by elution with the 6X-HIS elution buffer (50 mm phosphate buffer pH 7.0, 300 mm NaCl, and 150 mm imidizole). The GST signal of the candidate binding protein partners of 6X-HIS-IDDP10 was detected by SDS-PAGE with immunoblotting using a GST antibody (dilution 1:1000) (MA4-004-HRP, Thermo Fisher Scientific, Waltham, MA, USA).

### Gene ontology enrichment analysis

GO term enrichment analysis was performed at AgriGOv2 (http://systemsbiology.cau.edu.cn/agriGOv2/) with the Maize-GAMER (AGPv4) dataset used as the annotated background (Wimalanathan et al. 2018). The significant enrichment cutoff FDR was 0.05.

### Statistical analyses

All DNA sequencing readcount-related data in this study were normalized to fit the generalized linear model (GLM), and Empirical Bayes shrinkage estimation was used for calculating dispersions and FCs, as well as the *P*-values and the FDR (adjusted *P*-values). In addition, the interaction effects of NKD1, NKD2, and O2 on downstream genes were investigated via multifactorial experimental design. Two factors (2 of the 3 key TFs) and 2 levels (WT and mutant) were fitted in GLM to calculate the FC, and the FDRs were calculated via the BH procedure. Pearson correlation test was used to analyze the correlation between the expression of the key TF genes (*nkd1*, *nkd2*, and *o2*) and corresponding coexpression MEs. Significance levels were set to 0.05, 0.01, and 0.001.

Pairwise *t*-tests were used to compare the starch content, zein protein quantity, the normalized relative gene expression in selected modules between genotypes, and the relative nuclei number of the corresponding ploidy between WT and mutants. Significance level was set to *P* < 0.05. All pairwise *t*-tests were performed by JMP Pro 16 with default settings (SAS Institute).

Chi-square test was used to test if the observed phenotypic ratio among WT, *nkd1*, *o2*, and *nkd1 o2* agrees with the ratio of Mendel's law of segregation and to test if there is any *nkd1–o2* interaction. The threshold for significance was set to *P* < 0.05. If the corresponding probability level >0.05, we accept the null hypothesis that the data fit the expected ratio.

Hypergeometric tests were used to statistically evaluate if 2 groups of genes were significantly related by calculating the number of overlapping genes relative to the total number of protein-coding genes in the maize B73 RefGen_v4 genome (Jiao et al. 2017). The tests were performed as described (Zhan et al. 2015) and visualized as heatmaps that were built by the R package gplots (https://github.com/talgalili/gplots). The *P*-values were transformed by −log10p.

The statistical data are provided in Supplemental Data Set 21.

### Accession numbers

The *nkd1* gene corresponds to Zm00001d002654 in genome assembly B73 RefGen_v4 or Zm00001eb073830 in B73 RefGen_v5, *nkd2* corresponds to Zm00001d026113 or Zm00001eb428940, respectively, and o2 is Zm00001d018971 or Zm00001eb301570, respectively. Key hub gene accessions are shown in Table 2. The RNA-seq and ATAC-seq data of *nkd1*, *nkd2*, and *o2* single, double, and triple mutants, as well as DAP-seq data of TFs have been deposited in Gene Expression Omnibus (GEO), with the accession numbers GSE174059 (RNA-seq), GSE214415 (ATAC-seq), and GSE214166 (DAP-seq), respectively.

## Supplementary Material

koad247_Supplementary_Data

## Data Availability

All sequencing data have been deposited in Gene Expression Omnibus (GEO), with the accession numbers GSE174059 (RNA-seq), GSE214415 (ATAC-seq), and GSE214166 (DAP-seq). All other data required to evaluate conclusions is included in the paper or in Supplemental Data.

## Supplemental data

The following materials are available in the online version of this article.

**Supplemental Figure S1.** Kernel phenotypes of WT and *nkd1, nkd2*, and *o2* mutant combinations.

**Supplemental Figure S2.** Light micrographs of AL, SA, and SE from 2 plants each of the 8 genotypes.

**Supplemental Figure S3.** Electron micrographs of AL, SA, and SE for select genotypes (scale bar = 10 *μ*m).

**Supplemental Figure S4.** Protein body phenotype and size for selected genotypes.

**Supplemental Figure S5.** Interaction effects of *nkd1*, *nkd2*, and *o2* on nuclear ploidy.

**Supplemental Figure S6.** Number of DEGs in each mutant compared to WT at 8, 12, and 16 DAP.

**Supplemental Figure S7.** The distribution of potential NKD consensus binding motifs.

**Supplemental Figure S8.** Binding motifs of GBF1, HSFTH10, and corresponding networks.

**Supplemental Figure S9.** Genomic distribution of ATAC-seq (assay for transposase accessible chromatin with sequencing) peaks in the 8 genotypes.

**Supplemental Table S1.** Kernel phenotype statistics on +/*nkd1*, +/*o2* self-crossed segregating ears, and chi-square test for *nkd1-o2* interaction.

**Supplemental Table S2.** Pooled timepoint WGCNA module size.

**Supplemental Table S3.** Number of potential NKD1 and NKD2 targets and percentage of targets annotated as TFs at 8, 12, and 16 DAP.

**Supplemental Table S4.** Motif enrichment analysis of differential peaks overlapping with NKD2 target regions.

**Supplemental Table S5.** Primers and adapters.

**Supplemental Data Set 1.** DEGs mutant vs. WT.

**Supplemental Data Set 2.** Module gene content for pooled timepoint WGCNA.

**Supplemental Data Set 3.** GO terms for pooled timepoint WGCNA modules.

**Supplemental Data Set 4.** Module gene content for individual timepoint WGCNA at 8 DAP.

**Supplemental Data Set 5.** Module gene content for individual timepoint WGCNA at 12 DAP.

**Supplemental Data Set 6.** Module gene content for individual timepoint WGCNA at 16 DAP.

**Supplemental Data Set 7.** GO terms for individual timepoint WGCNA modules.

**Supplemental Data Set 8.** Interactions among *nkd1*, *nkd2*, and *o2* on gene expression.

**Supplemental Data Set 9.** NKD1-bound DNA sites by DAPseq.

**Supplemental Data Set 10.** NKD2-bound DNA sites by DAPseq.

**Supplemental Data Set 11.** GBF1-bound DNA sites by DAPseq.

**Supplemental Data Set 12.** HSFTF10-bound DNA sites by DAPseq.

**Supplemental Data Set 13.** Differentially accessible chromatin between *nkd1* vs. WT by ATAC-seq.

**Supplemental Data Set 14.** Differentially accessible chromatin between *nkd2* vs. WT by ATAC-seq.

**Supplemental Data Set 15.** Differentially accessible chromatin between *nkd1 nkd2* vs. WT by ATAC-seq.

**Supplemental Data Set 16**. Differentially accessible chromatin between *nkd1 o2* vs. WT by ATAC-seq.

**Supplemental Data Set 17.** Differentially accessible chromatin between *nkd2 o2* vs. WT by ATAC-seq.

**Supplemental Data Set 18.** Differentially accessible chromatin between *nkd1 nkd2 o2* vs. *o2* by ATAC-seq.

**Supplemental Data Set 19.** Differentially accessible chromatin between *o2* vs. WT by ATAC-seq.

**Supplemental Data Set 20.** Differentially accessible chromatin between *nkd1 nkd2 o2* vs. *nkd1 nkd2* by ATAC-seq.

**Supplemental Data Se 21.** Statistical analyses.

## Dive Curated Terms

The following phenotypic, genotypic, and functional terms are of significance to the work described in this paper:

GBF1 Gramene: AT4G36730
GBF1 Araport: AT4G36730

OHP2 Gramene: AT1G34000
OHP2 Araport: AT1G34000

BZIP17 Gramene: AT2G40950
BZIP17 Araport: AT2G40950

dna Gramene: EO:0007532
dna Araport: EO:0007532

ARF1 Gramene: AT1G59750
ARF1 Araport: AT1G59750

GEO Gramene: https://www.ncbi.nlm.nih.gov/geo/
GEO Araport: https://www.ncbi.nlm.nih.gov/geo/

VAL2 Gramene: AT4G32010
VAL2 Araport: AT4G32010
