# Supplementary material for: NAKED ENDOSPERM1, NAKED ENDOSPERM2, and OPAQUE2 interact to regulate gene networks in maize endosperm development
Source: Plant Cell. 2023 Oct 5;36(1):19–39. doi: 10.1093/plcell/koad247 (PMC10734603; doi:10.1093/plcell/koad247)
Supplement: koad247_Supplementary_Data [file koad247_supplementary_data.zip › tpc.23.00310Supplemental Figures and Tables.pdf]

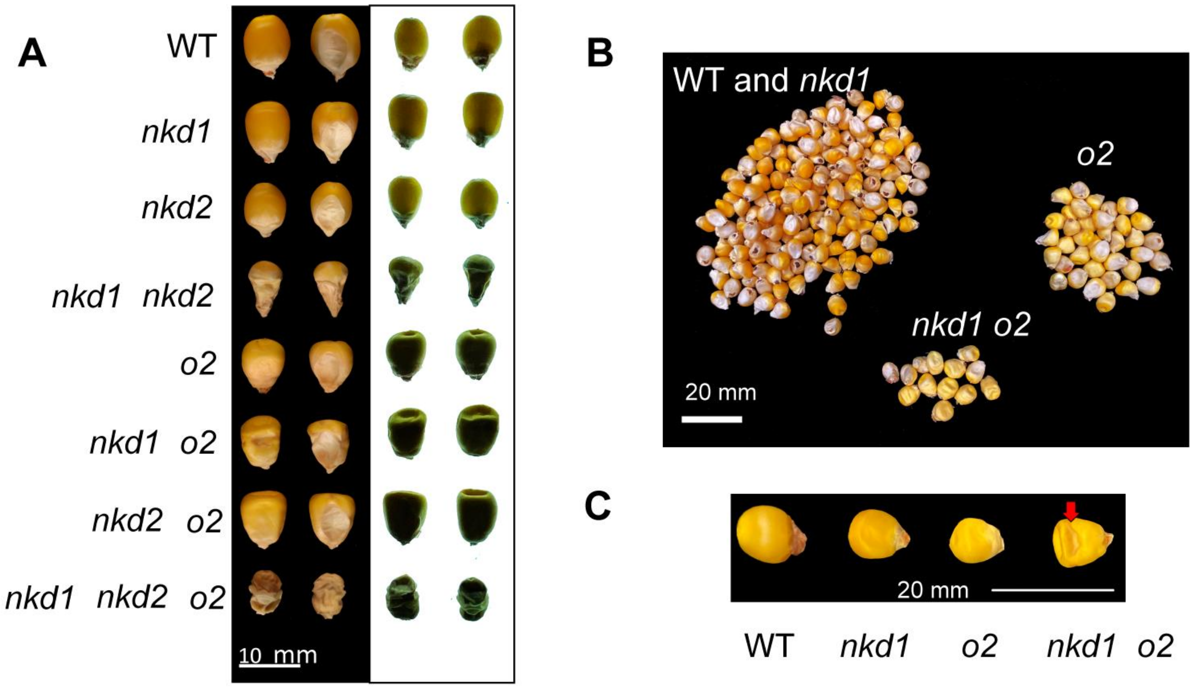

Supplemental Fig. S1. Kernel phenotypes of WT and *nkd1, nkd2* and *o2* mutant combinations. (Supports Figure 1A).

A. Kernel phenotype of the 8 genotypes. Black background: kernel surface overlook; White background: kernel opaqueness.

B. All kernels from a segregating ear of WT, *nkd1*, *o2* and *nkd1 o2*

C. Close look of the kernel phenotype of WT, *nkd1*, *o2* and *nkd1 o2*. Red arrow marks the phenotype of *nkd1 o2*.

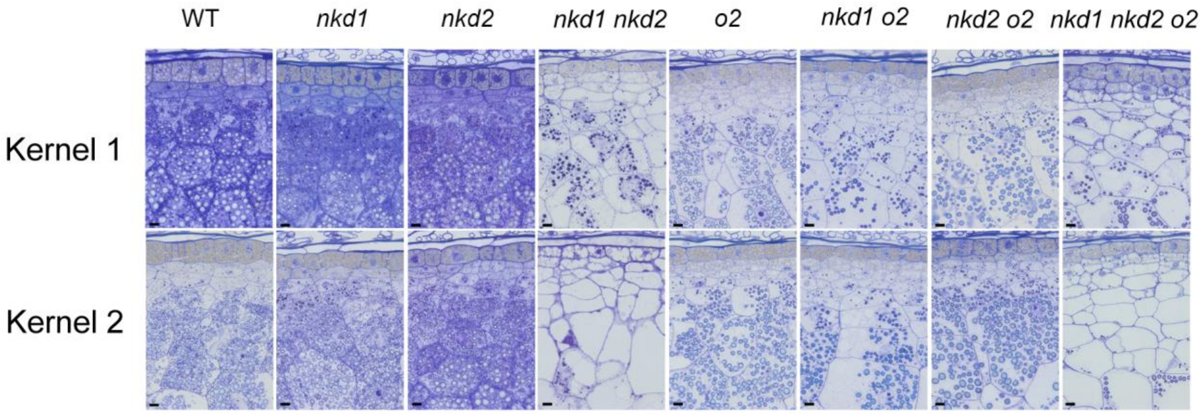

Supplemental Fig. S2. Light micrographs of aleurone, subaleurone and starchy endosperm from two plants each of the 8 genotypes. (Supports Figure 1B). Kernels 1 and 2 were dissected from independent ears. Scale bar = 10 µm.

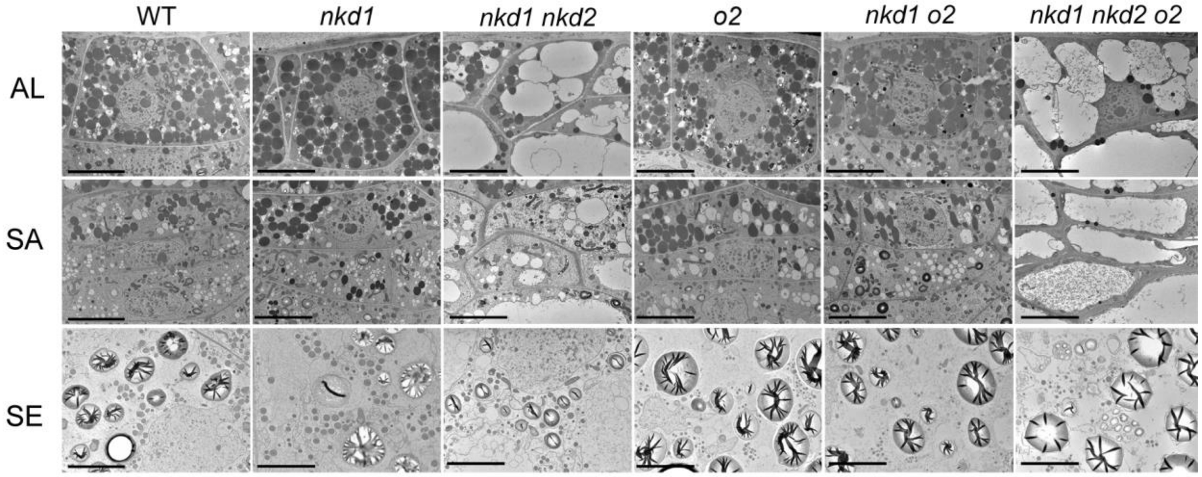

Supplemental Fig. S3. Electron micrographs of aleurone (AL), subaleurone (SA) and starchy endosperm (SE) for select genotypes. (Supports Figures 1C, D) Scale bar = 10 µm.

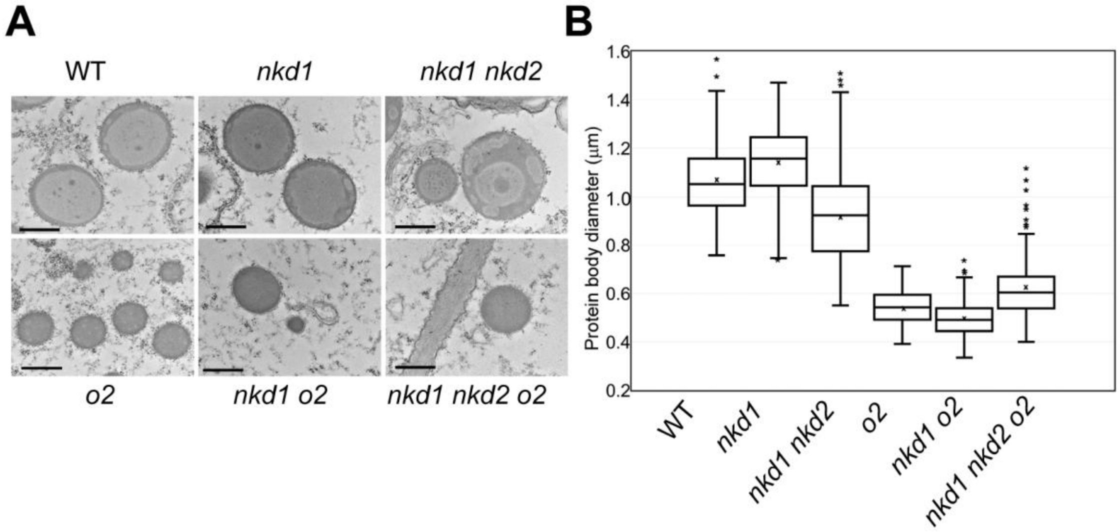

Supplemental Fig. S4. Protein body phenotype and size for selected genotypes. (Supports Figure 1D).

A. Transmission electron micrographs (TEM) of protein bodies in starchy endosperm cells. Scale bar = 0.5 μm

B. Quantitative comparison of protein body diameter. Component of the box plot: center line= median, box limits= upper and lower quartiles, whiskers= 1.5x interquartile range, cross mark=mean, asterisk= outliers. N ≥171 per genotype.

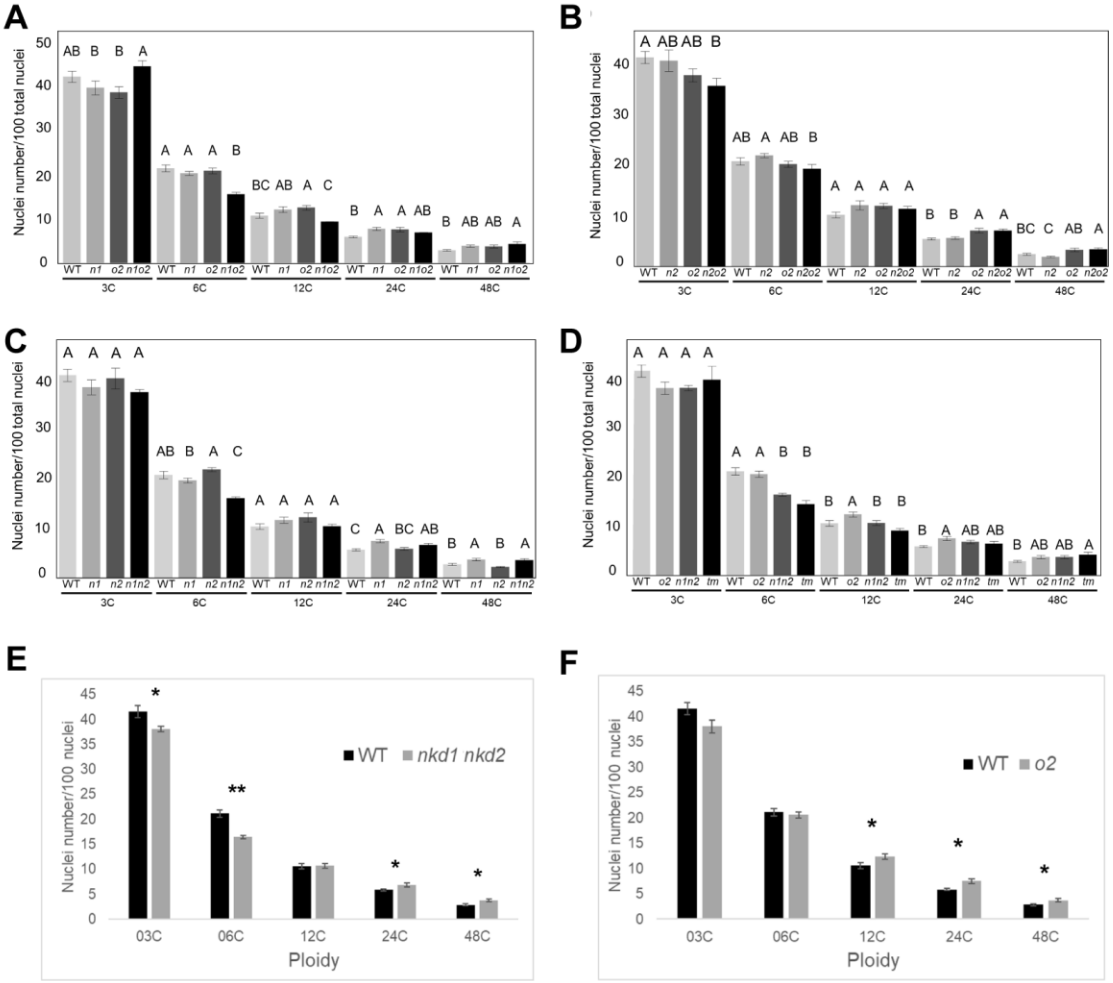

Supplemental Fig. S5. Interaction effects of *nkd1*, *nkd2* and *o2* on nuclear ploidy. (Supports Figures 1G and 8). Relative number of nuclei (nuclei number per 100 total nuclei) of 3C, 6C, 12C, 24C and 48C were compared between:

(A) WT, *nkd1*, *o2* and *nkd1 o2*

(B) WT, *nkd2*, *o2* and *nkd2 o2*

(C) WT, *nkd1, nkd2* and *nkd1 nkd2*

(D) WT, *nkd1 nkd2*, *o2* and *nkd1 nkd2 o2*

(E) WT and *nkd1 nkd2*

(F) WT and *o2.* Tukey's HSD test (A-D) or pairwise t-test (E and F) were used for comparisons. The error bars represent the standard error of 4 biological replicates from two independent ears of each genotype, two replicates per ear, each replicate contained a pool of three endosperms. The letter(s) and the single asterisk above the error bars represent the significance level by

$p<0.05$, and the double asterisk represents $p<0.01$.

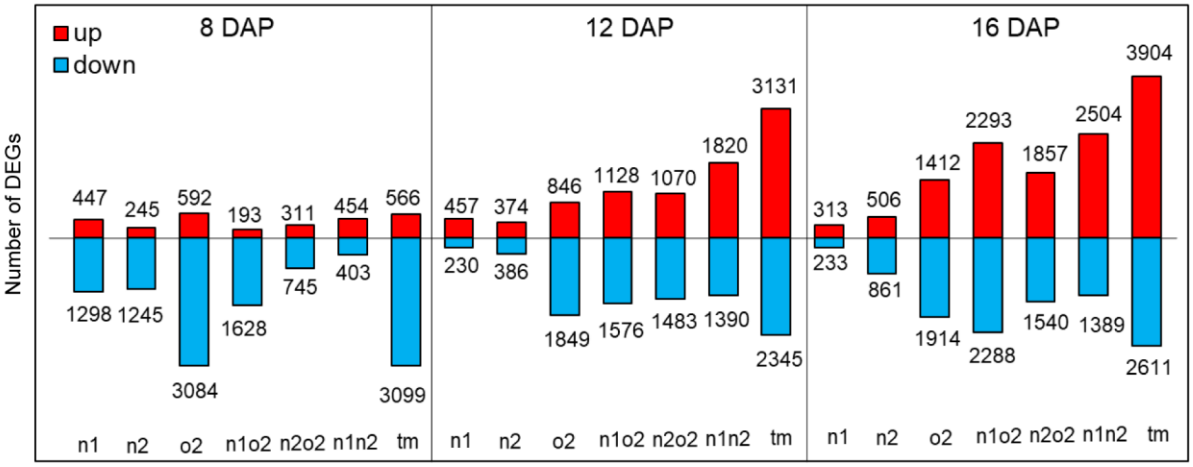

Supplemental Fig. S6. Number of differentially expressed genes (DEGs) in each mutant compared to WT at 8, 12 and 16 days after pollination (DAP). (Supports Figures 2 and 4). Red and blue bars represent numbers of up- and down-regulated genes, respectively, in the corresponding mutant versus WT comparision. The label n1, n2, o2, n1o2, n2o2, n1n2 and tm represent *nkd1*, *nkd2*, *o2* single mutants, *nkd1 o2*, *nkd2 o2*, *nkd1 nkd2* double mutants and *nkd1 nkd2 o2* triple mutant, respectively.

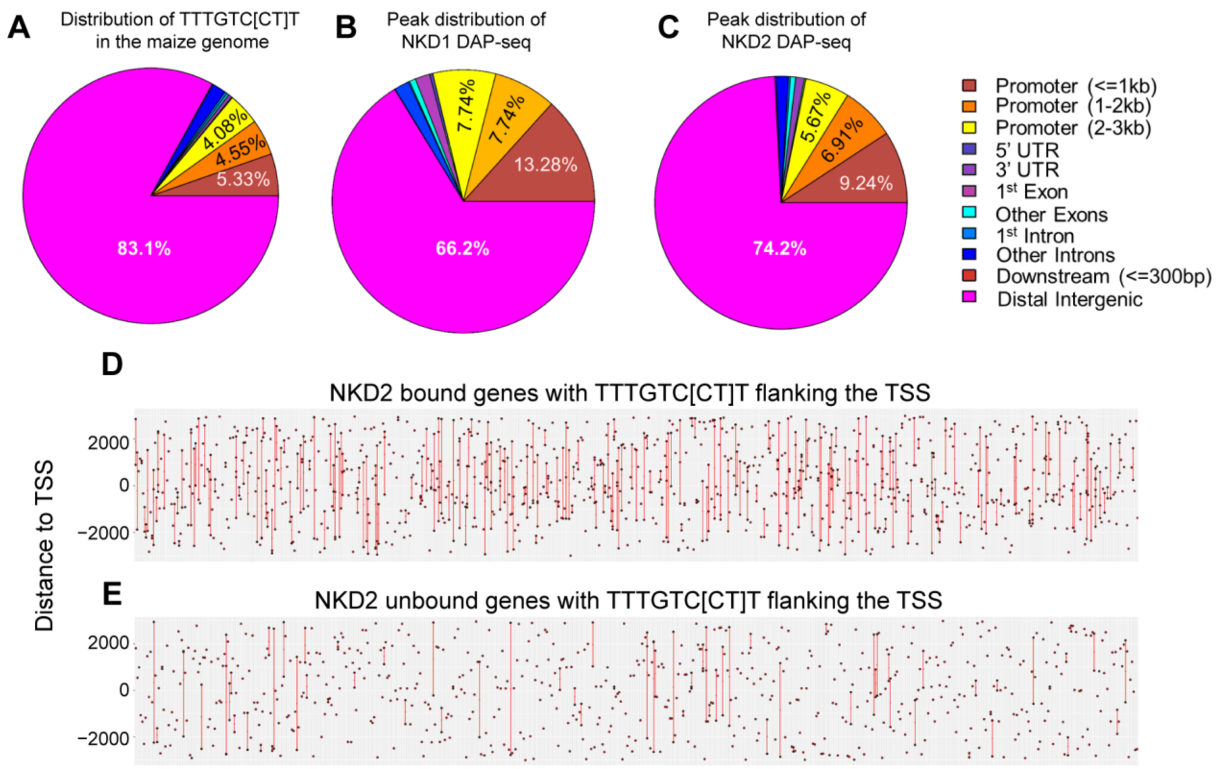

Supplementary Fig. S7. The distribution of potential NKD consensus binding motifs. (Supports Figure 6).

A. The distribution of the potential NKD consensus binding motif TTTGTC[CT]T in the maize genome.

B and C. DAP-seq peak distribution of NKD1 (B) and NKD2 (C).

D and E, NKD2 bound (D) and unbound (E) genes with the consensus binding motif flanking the annotated transcription start site (TSS) (-3000 bp to 3000 bp). 600 genes were randomly selected for each. Each red line connecting two or more dots represents a gene with two or more consensus motifs flanking the TSS, whereas a single dot represents a gene with only one consensus motif flanking the TSS. DAPseq=DNA affinity purification with sequencing.

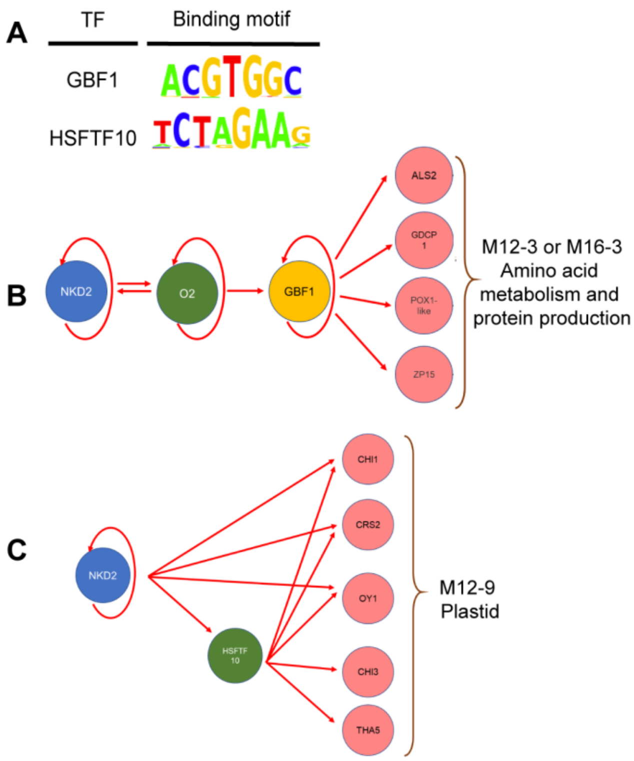

Supplemental Fig. S8 Binding motifs of GBF1, HSFTH10, and corresponding networks. (Supports Figures 6 and 8).

A. Binding motifs of GBF1 and HSFTF10.

B. Hierarchical network of NKD2-O2-GBF1-potential targets

C. Hierarchical network of NKD2- HSFTF10-GBF1-potential targets

Abbreviations: TF=transcription factor, M12-3=Module12-3 (so as M16-3 and M12-9), *GBF1= G-BOX BINDING FACTOR1, HSFTF10=HEAT SHOCK FACTOR TRANSCRIPTION FACTOR10*, *ALS2= ALUMINUM SENSITIVE 2, GDCP1= GLYCINE DECARBOXYLASE1, POX1-like= PROLINE DEHYDROGENASE 1-LIKE, ZP15=ZEIN PROTEIN 15, CHI1= CHALCONE-FLAVANONE ISOMERASE1* , *CHI3= CHALCONE-FLAVANONE ISOMERASE3, CRS2= CHLOROPLAST RNA SPLICING PROTEIN2* and *OY1=OIL YELLOW1.*

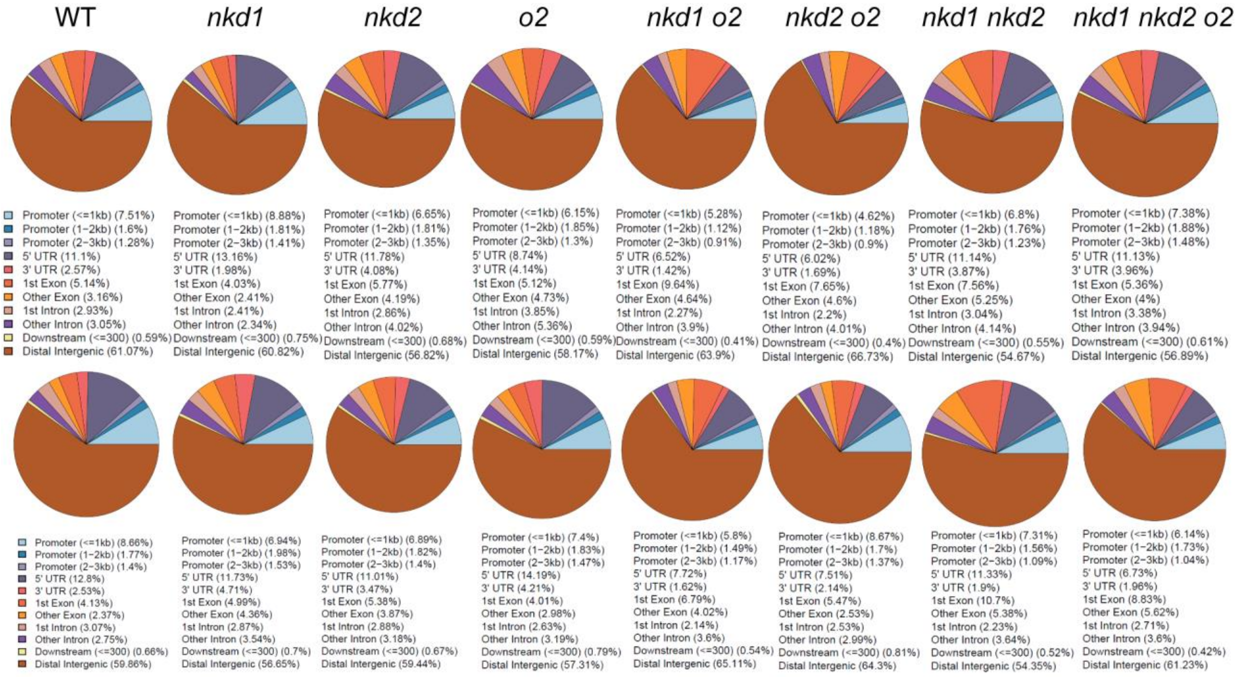

Supplemental Fig. S9 Genomic distribution of ATAC-seq (assay for transposase accessible chromatin with sequencing) peaks in the 8 genotypes. (Supports Figure 7). Row1: replicate#1, Row2: Replicate#2. Each replicate represented an independent ear from which 3 endosperms were dissected and pooled. Abbreviations: WT=wildtype, UTR=untranslated region.

Supplemental Table S1. Kernel phenotype statistics on +/*nkd1*,+/*o2* self-crossed segregating ears and Chi-square test for *nkd1-o2* interaction.

Ear#	**WT/*nkd1***		***o2***		***nkd1,o2***		**Chi-sq**	***p***
	**obs**	**exp**	**obs**	**exp**	**obs**	**exp**		
32-1	157	155.25	37	38.81	13	12.93	0.1045	0.949
32-10	250	251.25	63	62.81	22	20.94	0.0605	0.970
32-9	121	114	24	28.5	7	9.5	1.7982	0.407

Supplemental Table S2. Pooled-timepoint WGCNA module size. See the detailed gene information in Supplemental Data Sets 2–7)

Module	Number of genes
M1	529
M2	1253
M3	3384
M4	3501
M5	100
M6	1116
M7	981
M8	240
M9	803
M10	344
M11	209
non-clustered	1639
Total	14099

Supplemental Table S3. Number of potential NKD1 and NKD2 targets and percentage of targets annotated as TFs at 8, 12 and 16 DAP.

TF	DAP	Total target	Number of TF targets	Percentage of targets are TF
NKD1	8	22	11	50.0%
NKD2	8	223	101	45.3%
NKD1	12	20	5	25.0%
NKD2	12	323	116	35.9%
NKD1	16	25	9	36%
NKD2	16	397	134	34%

Supplemental Table S4. Motif enrichment analysis of differential peaks overlapping with NKD2 target regions.

Mutant	Differential peaks	Binding motif of	Adjusted p-value
nkd1	up	IDD	1.91E-21
		HMG	8.38E-04
nkd1	down	IDD	2.01E-07
		bZIP	1.65E-03
nkd2	up	IDD	1.38E-16
		BHLH	2.11E-04
nkd2	down	IDD	4.27E-06
nkd1,2	up	IDD	1.27E-06
		HMG	5.95E-03
nkd1,2	down	IDD	7.21E-25
		HMG	8.04E-07
		DOF	9.90E-07
		RING-type	3.05E-05
		MADS	1.37E-04

Supplemental Table S5. Primers and adapters.

Name	Sequence	Use
NKD1-forward	attB1-ATGGCATCGAATTCATCGGC	pENTR/SD/D-TOPO cloning for DAPseq constructs
NKD1-reverse	attB2-TGGCATCCTGCCTCCGTT	pENTR/SD/D-TOPO cloning for DAPseq constructs

NKD2-forward	attB1-ATGATGGCGTCGAATTCACC	pENTR/SD/D-TOPO cloning for DAPseq constructs
NKD2-reverse	attB2-TGGCATCCTGCCTCCATT	pENTR/SD/D-TOPO cloning for DAPseq constructs
IDDP10_HIS_Forward	GTACTTCCAGCCATGGCCATGCAAGCCAGGGACACC	Cloning IDDP10 into pET34b vector for 6XHIS fusion
IDDP10_HIS_Reverse	GTGCGGCCGCAAGCTTTTGCATCCTGTCTCCAGTGAAGG	Cloning IDDP10 into pET34b vector for 6XHIS fusion
NKD1_pF3K_Forward	TATCCCCACCGCGCGATCGATGTCCCCTATACTAGGTTATTGG	Cloning into expression vector pF3K WG (BYDV) Flexi for GST fusion protein
NKD1_pF3K_Reverse	AGCTCGAATTCGTTTAAACTCATGGCATCCTGCCTCC	Cloning into expression vector pF3K WG (BYDV) Flexi for GST fusion protein
NKD2_pF3K_Forward	TATCCCCACCGCGCGATCGATGTCCCCTATACTAGGTTATTGG	Cloning into expression vector pF3K WG (BYDV) Flexi for GST fusion protein
NKD2_pF3K_Reverse	AGCTCGAATTCGTTTAAACTCATGGCATCCTGCCTCC	Cloning into expression vector pF3K WG (BYDV) Flexi for GST fusion protein
IDDP10_pF3K_Forward	TATCCCCACCGCGCGATCGATGGCATCCAATTCATCAGCGG	Cloning into expression vector pF3K WG (BYDV) Flexi for GST fusion protein
IDDP10_pF3K_Reverse	AGCTCGAATTCGTTTAAACTTAGTGGTGGTGGTGGTGG	Cloning into expression vector pF3K WG (BYDV) Flexi for GST fusion protein
