# Supplementary material for: NAKED ENDOSPERM1, NAKED ENDOSPERM2, and OPAQUE2 interact to regulate gene networks in maize endosperm development
Source: Plant Cell. 2023 Oct 5;36(1):19–39. doi: 10.1093/plcell/koad247 (PMC10734603; doi:10.1093/plcell/koad247)
Supplement: koad247_Supplementary_Data [file tpc23.00310Peer Review Repotr.pdf › tpc23.00310Peer Review Repotr.pdf]

NAKED ENDOSPERM1, NAKED ENDOSPERM2, and OPAQUE2 interact to regulate gene networks in maize endosperm development

Hao Wu, Mary Galli, Carla J. Spears, Junpeng Zhan, Peng Liu, Ramin Yadegari, Joanne M. Dannenhoffer, Andrea Gallavotti, Philip W. Becraft

Corresponding author: Philip W. Becraft (becraft@iastate.edu)

Review timeline:

**TPC2023-RA-00310**	Submission received:	April 1, 2023
	1st Decision:	June 3, 2023 *revision requested*
**TPC2023-RA-00310R1**	1st Revision received:	July 4, 2023
	2nd Decision:	Jule 31, 2023 *accept with minor revision*
**TPC2023-RA-00310R2**	2nd Revision received:	Aug. 4, 2023
	3rd Decision:	Aug. 14, 2023 *acceptance pending, sent to science editor*
	Final acceptance:	Sept. 16, 2023

REPORT: (The report shows the major requests for revision and author responses. Minor comments for revision and miscellaneous correspondence are not included. The original format may not be reflected in this compilation, but the reviewer comments and author responses are not edited, except to correct minor typographical or spelling errors that could be a source of ambiguity.)

TPC2023-RA-00310 1st Editorial decision – *revision requested* June 3, 2023

We have received reviews of your manuscript entitled "NAKED ENDOSPERM 1, NAKED ENDOSPERM 2 and OPAQUE 2 Promote Transition to Grain-filling in Maize Endosperm Development." Thank you for submitting your best work to The Plant Cell. The editorial board agrees that the work you describe is substantive, falls within the scope of the journal, and may become acceptable for publication, pending revision and potential re-review.

We ask you to pay attention to the following points in preparing your revision:

1) Some of the conclusions of the paper must be toned-down as DAP-seq, even when combined with RNA-seq, is a poor substitute for establishing in vivo binding, for example, by ChIP. This limitation must be kept in mind when discussing the results and their meaning.

2) The title of the manuscript should be reconsidered in the light of what are the main conclusions of the study.

The reviewers raised several other points that we would like to see carefully addressed in a revision. I should stress that we are reluctant to see manuscripts undergoing multiple rounds of revision and would be unlikely to offer you more than one chance to satisfy the reviewers.

-- Reviewer comments:

[Reviewer comments shown below along with author responses]

TPC2023-RA-00310R1 1st Revision received July 4, 2023

Reviewer comments and **author responses**:

<u>Reviewer #1:</u>

While genome-based DAP-seq is a really useful method for defining consensus binding sites for diverse transcription factors, the tendency in the paper to treat DAP-seq as a substitute for or equivalent to ChIp-seq is a category error.

DAP-seq measures in vitro binding to purified genomic DNA, so there is no information on chromatin accessibility except for DNA methylation if TF binding is methylation sensitive.

We made 3 attempts at producing antibodies to NKD1 and NKD2, including working with a commercial company, and attempted to express epitope tagged versions in transgenic plants. Unfortunately none of these were successful which is why we turned to DAPseq. We are well aware of the limitations of DAPseq and, therefore, focused on those genes that contained DAPseq peaks AND were DEG. We were careful to always describe such genes as 'putative' targets. We have replaced 'putative' with 'potential' or 'possible' throughout the manuscript to further emphasize the limitations of DAPseq.

Thus, as expected, only a small percentage of DAP-seq peaks are in the vicinity of genes even with the generous 3 kbp window used in this analysis. In my hands a cursory text search of the B73v5 genome with a rough NKD2 binding site consensus, TTTGTC[CT]T in both orientations (Fig 6A), yields 209,000 sites. At least 56,800 (27%) of those were recovered in statistically robust DAP-seq peaks. So, as expected, most of the information gained from DAP-seq is contained in the distribution of the consensus motif. Nevertheless, one can ask where the ~150,000 unbound consensus sites are distributed in the genome. Are they less likely to be in or near genes than the bound class? If yes, then the DAP-seq peak distribution arguably contains at least some additional information not captured by the consensus (e.g. clustering or spacing of binding sites, etc). In that case are contexts of unbound consensus sites distinguishable in some way from the bound sites? In other words, is it possible to refine the consensus by focusing on the functionally relevant subset of DAP-seq peaks associated with ATAC-seq peaks and DEGs?

The suggestion to compare the distributions of bound versus unbound sites is excellent, thank you! We used FIMO of the MEME suite to search NKD2 binding consensus sequences TTTGTC[CT]T in both orientations and found 225,434 sites, similar to the reviewer's result. Their distribution is shown in panel A below. Approximately 14% of these sites were within the 3kb promoter region of genes. Bound sites had a higher likelihood of being in promoters, 28.76% for NKD1 and 21.82% for NKD2. Further, comparing bound and unbound sites within promoters, bound sites had a higher propensity to be in promoters that contained 2 or more consensus binding sites. We speculate this might relate to the ability of the NKD factors to dimerize. Thus, while we did not refine the consensus sequence, we did gain important contextual information. We have added these results to the manuscript in the first paragraph of the section 'NKD1, 2 and O2 regulate co-expression modules directly or indirectly through hub TFs' and as Supplemental Fig. S7.

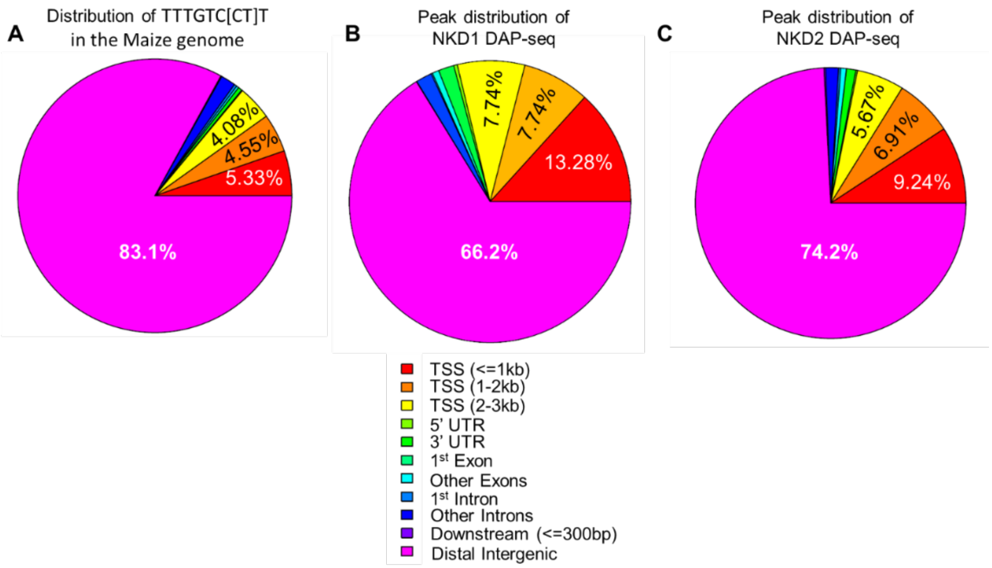

Relatedly, given the similarity of the NKD1 and NKD2 consensus sites, what accounts for the peak differences in Fig 7 A,C and G?

The reason for this is unclear. We have observed much better binding of NKD2 than NKD1 in several binding assays

using both in vitro transcribed and translated proteins as well as bacterially expressed proteins. We speculate that it is most likely a technical issue such as protein folding or the requirement for a co-factor.

The regulatory network (Fig 8) indicates that o2 inhibits endoreduplication. Is there flow cytometry evidence for increased endoreduplication in o2?

Yes, we present results of flow cytometry in Figure 1G and Supplemental Figure S5. The original Figure S5 tested interactions among different genes and showed a trend that *o2* mutant has fewer 3C nuclei than the WT, and more 12C and 24C nuclei than WT. We added a panel showing that in a direct comparison of *o2* mutant versus WT, all the higher order classes (above 6C) were statistically higher in *o2*. We also cite published work that shows increased DNA content in endosperm nuclei of *o2* mutants using Fuelgen staining and cytophotometry (Kowles and Phillips, 1985).

Line 591-93. What does O2+ signify? WT?

***O2*+ genotypes are defined as any genotype with homozygous WT *O2* allele, which refer to WT, *nkd1* or *nkd2* single mutant, and *nkd1;2* double mutant in the manuscript.**

Line 617 of Discussion, is auxin signaling implicated in M16-7, M16-2 or both? Fig 6 labels M16-2 as auxin related, whereas the ARF1 is associated with M16-7 in Table 2 which is labelled DNA replication/nucleosome assembly in Fig 6.

The functional annotations of the co-expression module are based on the GO term enrichment analysis. In M16-2, the auxin signaling genes are enriched so auxin signaling is implicated in M16-2, but in M16-7, genes associated with DNA replication/nucleosome assembly (endoreduplication-related) are enriched, even though *ARF1* may be involved in this process. In the paper Sabelli, 2012, auxin may function to promoting endoreduplication.

In the merged pdf image at least the smaller text and TF labels in Fig 6 are too fuzzy to be legible under zoom.

We uploaded images with higher resolution.

Fig 7G is mislabeled F.

Fixed.

The gene annotations should be updated to B7v5.

We used B73v4 due to the convenience of communication within the maize community and with researchers working on other plant species. Although B73v5 is the most recent version of maize genome, B73v3 and v4 are currently better-annotated with gene names, functions and GO terms. Most maize papers published recently were still using B73v3 or v4. B73v3 and v4 are still readily accessible at MaizeGDB, Grameme or other databases for ortholog comparisons and inferences. If we apply v5 accession numbers, it will be inconvenient as people most likely will convert them back to v3 or v4.

<u>Reviewer #3</u>:

About the title, I agree that the mutants in these three TFs affect the cellular development and grain-filling in endosperm, but current data did not support that they regulate the transition to grain-filling.

Thank you for the pointing this out. Phenotypic and transcriptomic data both suggest that 16DAP endosperms of the triple mutant have characteristics normally associated with earlier stages. Nonetheless, we agree that the previous title might have been an overinterpretation of the data and we have changed the title to: "NAKED ENDOSPERM 1, NAKED ENDOSPERM 2 and OPAQUE 2 Interact to Regulate Gene Networks in Maize Endosperm Development".

For the predicted target genes and network hub genes, some experimental validation using transient transactivation assay (e.g. BiLUC analysis in protoplast) could greatly enhance overall data quality.
The major aim of this study was to obtain a global perspective of the pathways, processes and networks that are regulated by NKD1, NKD2 and O2, and their interactions, and how these networks change over time during endosperm development. Given the scope of the study, such analyses as suggested by the reviewer would only be feasible for an insignificant number of potential target genes. We feel such detailed analyses would be more

appropriate in follow-up studies that focus on testing particular predictions made here.

The seedling DNA was used for DAP-seq analysis (p36, materials and methods), would this affect the prediction of binding profiles for TFs from endosperm?

While we cannot address this point directly, we believe available evidence suggests that DAPseq differences due to tissue source are likely to be relatively minor. Since DAP-seq uses purified genomic DNA, the only differences between seedling and endosperm DNA would be covalent modifications, such as methylation. We currently do not have any information on whether NKD binding is methylation sensitive and so that is an unknown. However, published data suggest differences between tissues would likely be relatively minor. As shown in our analysis below, comparing DNA methylation data from endosperm (Xu et al. Genome Biology (2022) https://doi.org/10.1186/s13059-022-02641-x) with leaves and shoot (Noshay et al. PLoS Genet. 2019 DOI: 10.1371/journal.pgen.1008291) revealed that methylation patterns are largely conserved between endosperm and leaves, with correlations of .83 and .84 for CG and CHG, respectively. This is comparable to the correlations of .87 and .88 between leaf and shoot. CHH methylation shows lower correlations, 0.39 between endosperm and leaf, but this is a minor form of methylation affecting only 1.82% of the endosperm genome, compared to 78.4 and 57.6% for CG and CHG, respectively (Lu et al., 2015, https://doi.org/10.1371/journal.pone.0139582). Further, in our extensive analysis of ARF binding using DAP-seq, we observed high reproducibility in binding sites using DNA from vegetative versus reproductive tissue sources, with correlation coefficients between 0.93 and .098 (Galli et al, 2018, Nature Communications, DOI https://doi.org/10.1038/s41467-018-06977-6). Taken together, it seems highly unlikely that using endosperm DNA would substantially change the global picture of NKD binding at the genome scale.

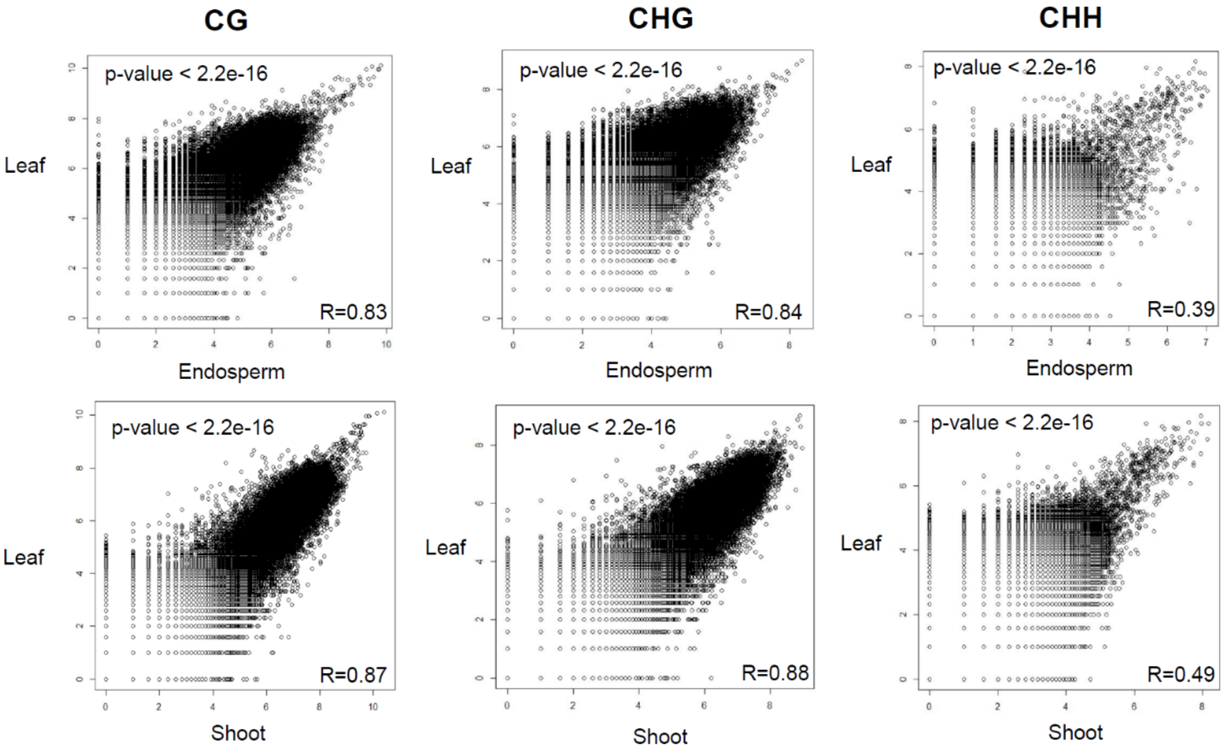

The data quality for NKD1 and 2 was not so balanced, with 1951 peaks for NKD1, and 56855 peaks for NKD2. This would affect the following target prediction and downstream analysis.

A reference for NKD1 and 2 putative binding targets is Gontarek et al., 2016 (Plant Cell, DOI: 10.1105/tpc.16.00609). The study identified 194 and 1050 target genes for NKD1 and 2, respectively, using selection and amplification binding (SAAB) techniques (see the Supplementary datasets). They also showed an "imbalanced" binding property between the two TF. The underlying mechanism or reason is unclear. The DNA binding property of in vitro transcribed and translated proteins could be influenced by multiple factors, such as technical pipelines, protein

folding, or the requirement of co-factors for binding, etc. Despite the difference between the number of target genes, DAP-seq in this study identified the binding consensus sequence similar to what identified in SAAB, but more specific to Maize genomic DNA and with higher sequencing quality than SAAB (because SAAB used randomly synthesized dsDNA, rather than maize genomic DNA). Therefore, the consistency of "imbalanced" number of identified target genes and consensus binding sequences between the two studies may suggest that our data could still be useful to perform target prediction and downstream analysis.

The DAP-seq and ATAC-seq data could be joint-analyzed for better prediction for TF targets.

We combined RNAseq and DAPseq to identify possible target genes. The ATACseq was based on 2 biological replicates (as is standard in the discipline), which provided very little statistical power for a meaningful joint analysis of all 3 datasets. Therefore, we selected several potential target genes to jointly analyze RNA-, DAP- and ATAC-seq data tracks. These are shown in Fig. 7. indicating that the differential chromatin accessibility supports the prediction of possible targets of NKD1 or/and NKD2 based on DEGs and DAP-seq.

The discussion part is too long, and could be significantly shorten.

We have shortened the discussion by 27%, from 2538 words to 1838. We agree the more succinct discussion is an improvement. We also removed most discussion of spatial gene expression and functions in response to Reviewer 4's concerns about such inferences.

Reviewer #4:

My major concern is that, although substantial amounts of work were executed in this manuscript, results obtained in this study did not go too much beyond what we have already known about these three TFs, nor advanced our understanding of the endosperm development in general. For example, the authors stated that "Genetic interactions in regulating this network are also dynamic. ATAC-seq analysis showed that O2 influences the global regulatory landscape, causing decreased accessible NKD1 and 2 target sites, while NKD1 and 2 increase accessible O2 sites.", how these processes are regulated by these TFs is still not clear. As such, the model proposed in Fig. 8 is purely based on expression and DAP-seq analyses, and chromatin accessibility profiling, lacking genetic and biochemical supports.

The main focus of this study is to investigate functional interactions among NKD1, NKD2 and O2 on regulating gene networks at the global scale from differentiation stage to grain-filling stage. Detailed mechanistic studies will be for follow-up work.

The study reported the following findings for the first time:

1. **NKD1, NKD2 and O2 function dynamically, regulating functions primarily associated with cellular development at early stages and transitioning to functions associated with nutrient accumulation and storage during grain filling. These functions were demonstrated at phenotypic and transcriptomic levels.**
2. **Gene co-expression modules were identified and associated with biological processes, along with hub genes including potential direct targets of NKD1, NKD2 and O2.**
3. **Effects of the 3 TFs (and their interactions) on chromatin accessibility were documented.**
4. **Genetic interactions were shown among *nkd1,nkd2* and *o2* at the phenotypic, transcriptomic, gene co-expression network, and epigenome levels.**
5. **The foundation of the study is a novel set of genetic materials that were bred for this analysis. The 3 mutant alleles were introgressed into an inbred background and then lines homozygous for the 3 mutations, in all combinations, were isolated.**

Regarding Fig.8, the model is indeed based largely on co-expression network, DAPseq and ATACseq analysis, and multifactorial analysis for interaction effects. However, the model is also supported by the following:

- **Eight genotypes cover all combinations of *nkd1*, *nkd2* and *o2* mutants as well as WT control. The diverse molecular, cellular and kernel phenotypes support genetic interactions among the three transcription factors.**

- **Our cellular studies showed that endosperm cells in *nkd1;nkd2;o2* triple mutant retain characteristics of cellular development stages at times when they should be in the nutrient accumulation stage, consistent with the model's proposal that NKD1, NKD2 and O2 may have important functions in this progression.**
- **Starch quantification, zein profiling, and TEM of protein bodies and lipid bodies (Fig. 1, Supplemental Fig. S1-S4) support the effects of NKD1, NKD2 and O2 on metabolism and accumulation of nutrient compounds.**
- **FACS showed a significant increase in nuclei with higher ploidy levels in the *o2* mutant as well as interactions with *nkd1* and *nkd2*.**
- **We previously published a study showing elevated expression of a DR5-RFP auxin reporter in AL cells of *nkd1;nkd2*, compared to WT, indicating that NKD1,2 may negatively regulate auxin signaling (Wu and Becraft, 2021. https://doi.org/10.1002/tpg2.20126).**

Other minor points:

1) In line 138, the authors stated that compared with WT, the nkd1;nkd2;o2 triple mutant has multiple layers of AL cells with multiple large vacuoles, fewer lipid bodies and irregular cell wall orientation (Fig. 1C), are those highly vacuolated cells in the lower panel of Fig. 1B really AL cells, or cells with delayed differentiation of AL and starchy endosperm? Detailed cell biological analyses of different staged endosperm are needed to make such statement.

> **Thanks for pointing this out. In previous studies of *nkd1;nkd2* mutants, we sometimes observed expression of aleurone marker genes in multiple layers of cells, and sometimes, loss of aleurone marker gene expression in the peripheral, would be aleurone, cells (Yi et al. 2015. https://doi.org/10.1104/pp.114.251413; Gontarek et al. 2016. https://doi.org/10.1105/tpc.16.00609). As such, it is hard to know what to call them. We have changed the wording in the text to 'poorly differentiated AL cells'. We left the labels in the figure for simplicity and consistency. The cells labeled AL in Fig. 1C correspond to cells from the same region of the endosperm labeled AL in Fig. 1B.**

2) In line 169, the authors stated that "The nkd1 mutant, alone or in combination with nkd2, o2, or both, tended to decrease the proportion of lower ploidy nuclei, particularly 6C, and increase the proportion of nuclei 24C and 48C (Fig. 1G; Supplemental Fig. S5, A, C and D). These data suggest that NKD1, NKD2 and O2 may function cooperatively to constrain endoreduplication." It will be very interesting to examine if the increased numbers 24C and 48C cells are in particular cell types in the maize endosperm, or a general change in all cell types in the endosperm. Is it possible that one of these three TFs may play a role of regulating endoreduplications in certain cell types?

> **From prior studies, endoreduplication mainly occurs in starchy endosperm cells of the WT. This study suggests that NDK1, NDK2 and O2 interactively regulate endoreduplication, but we have no direct information on the cell types involved.**

3) Expression and co-expression analyses suggest that "NKD1, 2 and O2 interact to regulate gene networks"

> **We are not sure we understand this comment. Perhaps a suggestion for an alternative title? If so, we have changed the title along these lines.**

4) In line 381, the authors stated that "DEGs of nkd1;nkd2 are associated with almost every cell type at every stage, whereas o2 DEGs have a much more limited role at 8 DAP, only associated with BETL and CSE (Fig. 5).", detailed analyses (such as in situ hybridization and reporter lines) are needed to confirm such a statement.

> **We agree. In this study, we mainly focus on *temporal* analyses of maize endosperm development (8-16 DAP). For the spatial (i.e. cell type) analysis, we analyzed the correlations between previously published LCM datasets and ours. The LCM data is from 8 DAP endosperm, so we feel that the comparison with our 8 DAP data is appropriate and provides some useful information. However, we agree that the correlations between the LCM and our 12 and 16 DAP data are not valid. As such, we have removed panels 5 B,C and any mention of spatial gene expression related to these later time points, as well as all mention of spatial gene expression patterns from the Discussion.**

TPC2023-RA-00310R1 2nd Editorial decision – *accept with minor revision* July 31, 2023

We have received reviews of your manuscript entitled "NAKED ENDOSPERM 1, NAKED ENDOSPERM 2 and

OPAQUE 2 Interact to Regulate Gene Networks in Maize Endosperm Development." On the basis of the advice received, the board of reviewing editors would like to accept your manuscript for publication in The Plant Cell. This acceptance is contingent on revision based on the comments of our reviewers. In particular, please consider the following:

• Lines 157 - 162: The sentence beginning with "The nkd1;nkd2 double mutants showed greater dark-staining areas with reduced,..." is partially duplicated - please check.

• Avoid double conditionals (...suggest that .... may) - example in lines 174, 357, 366

• Break in sentence in line 185, and void starting sentences with a number (spell it out, e.g., line 186 but other instances as well)

• Spell out O2+ the first time it is mentioned in the manuscript body as done in the rebuttal letter

• Line 211: change were shown to are shown

• A number of small corrections such as .- (line 305), missing spaces after brackets (multiple times), extra spaces before a comma, etc. A careful reading of the manuscript could catch most of these mistakes

• The manuscript talks about TSS (e.g., line 329) - about 50% of the TSS in B73 v4 are improperly annotated - please refer to TSS only when experimental evidence is available. Otherwise I suggest to refer to annotated TSS.

• In Fig. 3A, should NKD1, NKD2 and O2 be small case and in italics? Referring to the genes, no? Same for the title of Fig. 4 and legends in Fig. 5? Please check throughout.

• Please number the lanes in Fig. 6E - makes it easier to reference the figure. Legend for this figure should explain the figure better, not describe the conclusion.

-- Reviewer comments:

Reviewer #1:

The authors have responded satisfactorily to my comments and suggestions.

Reviewer #3:

In this revision, the authors had answered all my questions, and provided proper adjustment or reasonable explanation. I have no further question.

Reviewer #4:

No more comments.

TPC2023-RA-00310R2 2nd Revision received Aug. 4, 2023

Editor's comments and **author responses**:

• Lines 157 - 162: The sentence beginning with "The nkd1;nkd2 double mutants showed greater dark-staining areas with reduced,..." is partially duplicated - please check.

The duplication was removed, lines 160-162.

• Avoid double conditionals (...suggest that .... may) - example in lines 174, 357, 366

Double conditionals and redundancies (Additionally…also) were removed from lines 89, 174, 195, 359, 369, 505.

• Break in sentence in line 185, and avoid starting sentences with a number (spell it out, e.g., line 186 but other instances as well)

Breaks have been removed lines 185, 312.

Numbers starting sentences have been corrected lines 186, 206, 208, 324, 381-382,

• Spell out O2+ the first time it is mentioned in the manuscript body as done in the rebuttal letter

O2+ was defined lines 190-191. Likewise, NKD1+ and NKD2+ were defined line 193.

• Line 211: change were shown to are shown

Correction made (now line 213).

• A number of small corrections such as .- (line 305), missing spaces after brackets (multiple times), extra spaces before a comma, etc. A careful reading of the manuscript could catch most of these mistakes

Spaces and extraneous characters were removed or added as needed lines 52, 57, 59, 62, 65, 68, 71, 76, 79, 89, 90, 91, 95, 96, 98, 100, 153, 156, 183, 204, 218, 314, 324, 334, 344, 359, 368, 376, 397, 447, 496, 500, 502, 506, 507, 518, 547, 559, 564, 570, 597, 604, 614, 617, 621, 623, 637, 641, 669, 678, 681, 689, 708, 709, 711, 736, 740, 742, 748, 752, 759, 791, 796, 852.

• The manuscript talks about TSS (e.g., line 329) - about 50% of the TSS in B73 v4 are improperly annotated - please refer to TSS only when experimental evidence is available. Otherwise I suggest to refer to annotated TSS.

We now refer to "annotated TSS", lines 326, 330-332, 384, 385.

Similarly, "annotated gene" line 383.

• In Fig. 3A, should NKD1, NKD2 and O2 be small case and in italics? Referring to the genes, no? Same for the title of Fig. 4 and legends in Fig. 5? Please check throughout.

Corrected in Fig 3A, and lines 207, 243, 250, 452-454, 851, 857, 874, 878, 889-893.

• Please number the lanes in Fig. 6E - makes it easier to reference the figure. Legend for this figure should explain the figure better, not describe the conclusion.

The lanes have been labeled and the legend updated, lines 911-917.

Several other edits were made as follows:

Commas in *nkd1,2* were replaced with semicolons for consistency, lines 99, 133, 296, 297 300, 313, 391, 394, 395, 416, 418, 420, 427, 437, .

The notation of referring to *nkd1;nkd2* double mutants as *nkd1;2* is defined on line 73.

Line 81 citations were combined.

Line 171, an orphan call-out was removed.

Line 207 and 250, *O2* changed to *o2.* Line 243, *NKD1* changed to *nkd1.*

Line 255, in changed to at.

Line 307, sentences combined for readability.

Lines 351-353, rearranged sentence for readability.

Lines 356, 554 inserted missing words.

Line 399, 498 + signs inserted.

Lines 415, 421, 428, 465, 490, 673, 697, 715-717, 744, 797 sentences edited for readability.

A reference (Burdo et al., 2014) and link to the TFome stock center were added, line 652.

Line 662, degrees replaced with °C; line 668 C replaced with °C.

Line 667, seven replaced with 7.

TPC2023-RA-00310R2 3rd Editorial decision – *acceptance pending* **Aug. 14, 2023**

We are pleased to inform you that your paper entitled "NAKED ENDOSPERM 1, NAKED ENDOSPERM 2 and OPAQUE 2 Interact to Regulate Gene Networks in Maize Endosperm Development" has been accepted for publication in The Plant Cell, pending a final editorial review by a science editor.

Final acceptance from Science Editor **Sept. 16, 2023**
